# A modeling study on SARS-CoV-2 transmissions in primary and middle schools in Illinois

**DOI:** 10.1186/s12889-024-20623-5

**Published:** 2024-11-18

**Authors:** Conghui Huang, Rebecca Lee Smith

**Affiliations:** 1 Department of Pathobiology, College of Veterinary Medicine, University of Illinois at Urbana Champaign, Urbana, IL USA; 2 Carle-Illinois College of Medicine, University of Illinois at Urbana Champaign, Urbana, IL USA; 3 Institute of Genomic Biology, University of Illinois at Urbana Champaign, Urbana, IL USA

**Keywords:** Compartment model, Primary and middle schools, SARS-CoV-2 transmission, Within-school transmission

## Abstract

**Background:**

The global pandemic caused by the SARS-CoV-2 virus led to a statewide lockdown in Illinois starting in March 2020. To ensure students’ and employees’ safety for school reopening, protective measures, such as a statewide mask mandate and weekly testing, were in place in Illinois from Spring 2021 to Spring 2022. The study objective is to 1) estimate the in-school and external transmission of SARS-CoV-2 in elementary and middle schools under mask mandate and weekly surveillance and 2) estimate the impacts of protective measures such as testing and mask proportion and testing frequency on SARS-CoV-2 transmission.

**Methods:**

A stochastic compartmental model was built to simulate the SARS-CoV-2 transmission within and between the student and employee groups in primary and middle schools participating in the weekly testing program and to evaluate the effectiveness of these protective measures. This stochastic model was modified from a susceptible–infected–recovered framework and calibrated to SARS-CoV-2 surveillance data in 116 primary and middle school districts from Spring 2021 to March 2022. This model calibration was assessed using the surveillance data from the rest of the spring semester in 2022.

**Results:**

Overall, the external transmission rates in students and employees were significantly greater than those within schools, and the external transmission rates in middle school students and school employees were greater than those in primary school students. Our sensitivity analysis showed that transmission rates within student groups could significantly influence overall infection rates in vaccinated and unvaccinated students in large school districts. Under the protective measures implemented in the studied period in Illinois, an increased proportion of students and employees participating in the weekly testing can decrease infections. However, community-level measures of self-reported mask adherence among adults were not significantly associated with the infections during the study period, when a universal mask policy was in place for the state.

**Conclusions:**

Although increased testing proportion and/or frequency can reduce the SARS-CoV-2 infections, the costs of testing can increase with the testing volume. Further studies on the cost-effectiveness between the testing volume and cases reduction or learning disturbance can aid in policy development to reduce transmission effectively.

**Supplementary Information:**

The online version contains supplementary material available at 10.1186/s12889-024-20623-5.

## Introduction

The SARS-CoV-2 global pandemic led to a three-month closure for in-person instruction in most kindergarten to grade 12 (K-12) public school districts in the U.S. after the detection of community transmission in March 2020 [1]. Many school districts switched to online instructions after the closure [1]. However, multiple challenges were found during the switch to online instruction in primary and middle school districts in 2020 [1]. Challenges in the transition to distance learning during the pandemic were reported in the lack of preparedness and limited access to remote teaching, engagement and participation, communication with students, parent involvement and home environment, and limited resources in instructional support [2]. To ensure the quality of education, some school districts started to resume hybrid or in-person instruction in the Fall of 2020 and increased in-person instruction in mid-Spring 2021 [1]; by Fall of 2021, most public school districts in the U.S. had resumed primarily in-person instruction. However, while vaccinations became available for individuals of age greater than 16 years old at the beginning of 2021, vaccinations for students in primary and middle schools were not made available until much later [1]. The approval of vaccinations was extended to individuals 12–15 years old in May 2021 and to individuals older than 5 years old in October 2021 [1]. The proportions of fully vaccinated (at least receiving a two-dose vaccination) individuals in the age group of 5 to 17 and above 18 reached 29.9% and 52.3%, respectively, by the end of 2021 in Illinois, USA [3]. Therefore, a safe reopening plan in school districts was needed to minimize disruption of in-person instruction and protect employees and students, especially unvaccinated individuals, from secondary transmissions.

The reopening plans employed by public school districts included mitigation measures such as masking mandates, testing and screening policy, social distancing, and sanitation [4]. Masks can effectively reduce airborne transmission of viral aerosols, especially in indoor scenarios [5, 6]. Fabric masks can achieve at least 64% of viral filtration efficiency on aerosol with an average size of 2.6 µm, which can reach the low respiratory system [7]. Although surgical masks and N95 masks can achieve 99% of viral filtration efficiency on aerosols with similar average sizes, the supply of these masks was limited at the beginning of 2021. Studies have shown that indoor mask mandates were associated with a reduction in secondary transmission of SARS-CoV-2 in K-12 schools in 2021 [8, 9]. Routine screening and symptom-based testing programs have been shown to identify and mitigate SARS-CoV-2 infections in students and employees [4]. However, the effectiveness of mask mandates and testing programs in decreasing in-school or/and community-based transmission has not been thoroughly quantified and validated using available testing results. Additionally, estimation of the effectiveness of mask mandates and testing programs can be challenging because of the heterogeneity and complexity of resources and demographics in public school districts.

In this study, we used aggregated results of screening and diagnostic testing in 116 public school districts with primary schools and middle schools participating in a weekly testing program to develop and parameterize a stochastic compartment model for SARS-CoV-2 transmission under indoor mask mandates and weekly voluntary testing programs for students and employees. We used this fitted model to determine the factors driving incidence risks in students and employees based on a global sensitivity analysis. We also compared the simulated incidence risks under different testing scenarios to assess the relative value of testing programs.

## Methods

### School district characteristics

**Fig. 1:**
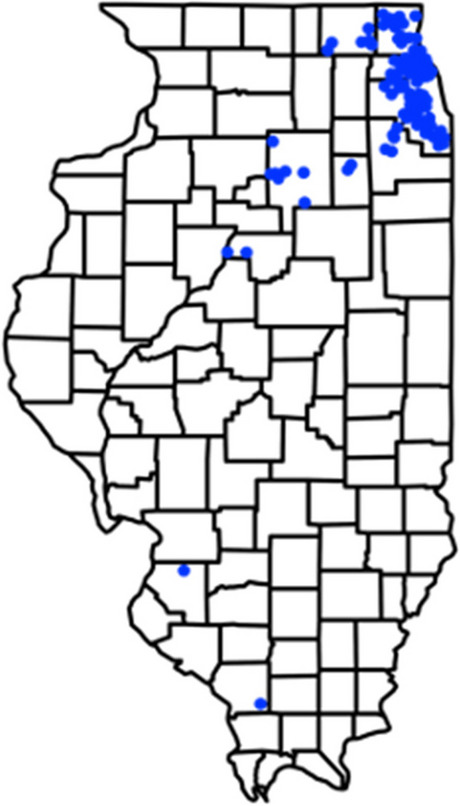
Primary and middle school districts (solid blue circles) participating in weekly SARS-CoV-2 surveillance testing at any point between January 2021 and May 2022 in Illinois, US

School testing data were provided by Shield Illinois (ShieldIL), which conducted weekly in-school testing on behalf of the Illinois Department of Public Health (IDPH) for all participating schools in the state excluding Chicago Public Schools. The school districts participated in the weekly testing program was shown in Fig. 1. Based on the school calendar in Illinois in the studied period [10], we divided the duration of the data collection into semesters as follows: Spring 2021 (January 5, 2021, to May 30, 2021), Fall 2021 (August 22, 2021, to December 22, 2021), and Spring 2022 (January 4, 2022, to May 28, 2022). Cumulative vaccination proportions within each school district and age group (students and employees) were estimated by the number of vaccinated individuals reported by the IDPH [3] and normalized by the county-level population in the specific age groups as reported by the 2022 U.S. Census Bureau [11]. The populations and proportions of students and employees in the studied school districts are reported by Elementary/Secondary Information System (ElSi) database [12], as shown in Table 1. This study was ruled exempt by the University of Illinois Institutional Review Boards (protocol #22843).

**Table 1:** Characteristics of school districts participating in the weekly SARS-CoV-2 surveillance testing at any point between January 2021 and May 2022 in Illinois, USA

Proportion of School Population (95% percentile)			Median total population (95% percentile)
Primary school student proportion (K-5th) (95% percentile)	Middle school student proportion (6th-8th grade) (95% percentile)	Employee proportion (95% percentile)	
0.55 (0.51–0.59)	0.31 (0.28–0.35)	0.13 (0.10–0.18)	1591 (216–6535)

### Model setup

**Fig. 2:**
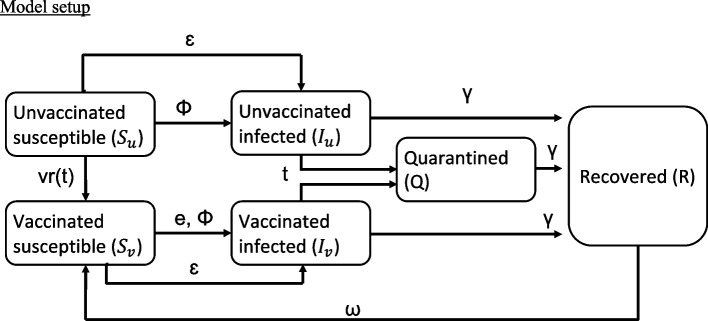
Schematic of the compartment-based SIR model for SARS-CoV-2 transmission in primary and middle schools. Parameters are defined in Table 2

SARS-CoV-2 transmission in primary and middle schools in Illinois from Spring 2021 to Spring 2022, was simulated based on a stochastic compartmental model following a susceptible-infected-recovered structure (Fig. 2). Specifically, susceptible individuals in the schools (${S}_{u}$ and ${S}_{v}$) were infected after in-contact with an infected individual within the school or in the community. The transmission rates of SARS-CoV-2 within school or in the community were simulated by $\Phi$ and $\varepsilon$, respectively. As vaccination became available in the beginning of 2021, unvaccinated individuals completed the two-dose mRNA vaccination at a rate of $v{r}_{i}(t)$. An infected individual (${I}_{u}$ and ${I}_{v}$) could be detected by a weekly test, based on the proportion of the school being tested in each week. A saliva-based quantitative real-time polymerase chain reaction (RT-qPCR) assay approved under the Emergency Use Authorization (EUA) issued by the U.S. Food and Drug Administration (FDA) (covidShield) was used in the weekly testing program. An infected individual who received a confirmed RT-qPCR test result entered a five-day quarantine as required by CDC (Q); those who were undetected remained in the school and in contact with the rest of the school population. The infected or quarantined individuals were assumed to recover (R) at rate γ. Recovered individuals were assumed to become susceptible again at rate $\omega$, reflecting the loss of immunity over time. We assumed that recovered individuals remained partially immune to infection, similar to the two-dose vaccine efficacy. The rates of change between states in student or employee are simulated using the equations [1] to [6]. N is the total school district population.

1 $$\frac{{dS}_{u}}{dt} =- vr\left(t\right) \times {S}_{u}\left(t\right) - {\alpha }_{wave} \times \sum \left[{\varnothing }_{k} \times {S}_{u,j} \left(t\right) \times \frac{{I}_{v,j} \left(t\right) + {I}_{u,j} \left(t\right)}{N - \sum {Q}_{j}\left(t\right)} + {\varepsilon }_{j} \times c\left(t\right) \times {S}_{u,j} \left(t\right)\right]$$

2 $$\frac{{dI}_{u}}{dt} ={\alpha }_{wave}\times \sum \left[{\varnothing }_{k} \times {S}_{u,j} \left(t\right) \times \frac{{I}_{v,j} \left(t\right) + {I}_{u,j} \left(t\right)}{N - \sum {Q}_{j}\left(t\right)} + {\varepsilon }_{j} \times c\left(t\right) \times {S}_{u,j} \left(t\right)\right] -\upgamma \times {I}_{u}\left(t\right) -t\times {I}_{u} \left(t\right)$$

3 $$\frac{{dS}_v}{dt}=vr\left(t\right)\times S_u\left(t\right)-\alpha_{wave}\times\sum\left[\varnothing_k\times S_{v,j}\left(t\right)\times\frac{I_{v,j}\left(t\right)+I_{u,j}\left(t\right)}{N-\sum Q_i\left(t\right)}+\varepsilon_j\times c\left(t\right)\times S_{v,j}\left(t\right)\right]+w\times R\left(t\right)$$

4 $$\frac{{dI}_{v}}{dt} ={\alpha }_{wave}\times \sum \left[\left(1 - {e}_{i}\right) \times {\phi }_{k}{S}_{v,j}\left(t\right) \times \frac{{I}_{v,j} \left(t\right) + {I}_{u,j} \left(t\right)}{N - \sum {Q}_{j}\left(t\right)} + {\varepsilon }_{j} \times c\left(t\right) \times {V}_{j} \left(t\right)\right] -\upgamma \times {I}_{v}\left(t\right) -t\times {I}_{v} \left(t\right)$$

5 $$\frac{dQ}{dt} =t\times \left({I}_{u} \left(t\right) + {I}_{v} \left(t\right)\right) - {\upgamma }_{q} \times Q\left(t\right)$$

6 $$\frac{dR}{dt} =\upgamma \times \left({I}_{u}+ {I}_{v}\right) + {\upgamma }_{q} \times Q\left(t\right) -w \times R\left(t\right)$$

### Model parameterization

#### Initialization

The total population in each school was initialized based on the school populations reported by U.S. Department of Education [12] and the county-level incidence in Illinois, USA [13]. The initial numbers of infected individuals the schools at the beginning of the spring and fall semesters were approximated by a multinomial distribution based on the school demographic information [12], daily laboratory-confirmed SARS-CoV-2 cases and normalized by county-level population in each county (c_i_(t)) reported by CDC [13], and seroprevalence of SARS-CoV-2 reported in a national commercial lab survey (${r}_{i}(t)$) by CDC [14]. The parameters were shown in Table 2. The county-level community incidence and state-level seroprevalence were categorized in two age groups (below and above 19 years old), which corresponded to students and employees in the studied primary and middle school districts. The model school populations were constructed by a multinomial distribution with total populations (P_total_) and proportions of students in primary (P_s1_) and middle schools (P_s2_) and employees (P_e_) (Multi(P_total_, P_s1_, P_s2_, P_e_)). The populations and proportions of students and employees were randomly drawn from the studied school districts, as shown in Table 1. The numbers of susceptible, infected, and recovered students or employees were estimated by a multinomial distribution (Multi(P_j_, 1-(c_i_(t) + ${r}_{i}(t)$),), when j = s1 or s2, i = student; j = e, i = employee). The vaccination status among the susceptible and infected individuals was estimated by a Poisson distribution with a *λ* consisting of the product of the total school population and a county-level vaccination rate (v_i_ (t), when i = student or employee) obtained from the CDC when t is the beginning of the school semester [3]. The masked populations in students and employees were simulated by a Poisson distribution with a *λ* of mask adherence (${m}_{i}(t)$, i indicates student or employee group) obtained from a nationwide survey on COVID-19 trends and impact [15], and the testing population was simulated based on the weekly number of tests included in the surveillance data obtained from the studied schools, respectively (Table 2). The model time step was one day.

**Table 2:** Model parameters used in the simulations

	Parameter	Distribution/Range	Sources
Model parameters			
Transmission among students in kindergarten to 5th grade	${\beta }_{ss1}$	Min = 0, max = 1.2	Derived
Transmission among students in 6th to 8th grade	${\beta }_{ss2}$	Min = 0, max = 1.2	Derived
Transmission between employee and students in kindergarten to 5th grade	${\beta }_{se1}$	Min = 0, max = 1	Derived
Transmission between employee and students in 6th to 8th grade	${\beta }_{se2}$	Min = 0, max = 1	Derived
Transmission among employees	${\beta }_{ee}$	Min = 0, max = 1.2	Derived
Loss of immunity rate	$\omega$	Min = 0, max = 1	Derived
Recovery rate of quarantined individual	$1/{\gamma }_{q}$	5	[16]
Recovery rate of infected individual	${1/\gamma }_{i}$	Triangular (First wave: min = 4.7, mode = 5.5, max = 6.5; second and third wave: min = 4.1, mode = 4.7, max = 5.6)	[17, 18]
Transmission between masked and unmasked individual	${\delta }_{mu}$	Min = 0.44, max = 1	Derived
External transmission to students in kindergarten to 5th grade	${\varepsilon }_{s1}$	Min = 0, max = 3	Derived
External transmission to students in 6th to 8th grade	${\varepsilon }_{s2}$	Min = 0, max = 3	Derived
External transmission to employee	${\varepsilon }_{e}$	Min = 0, max = 3	Derived
Infectivity of first wave	${\alpha }_{1}$	Min = 1, max = 3.5	Derived
Infectivity of second wave	${\alpha }_{2}$	Min = 1, max = 3.5	Derived
Infectivity of third wave	${\alpha }_{3}$	Min = 1, max = 5	Derived
Mask efficacy based on contact scenarios	${\delta }_{l}$	${\delta }_{mm}$=0.44;${\delta }_{uu}=1$;${\delta }_{um}$= [${\delta }_{mm}$,${\delta }_{uu}$]	[19] or Derived
Vaccine efficiency	e	Triangular	[20]
qPCR test sensitivity and specificity	t	99%	[21]
Time-dependent parameters			
Vaccination rate	vr_i_ (t)	i indicated student or employee	[3]
Vaccination proportion	v_i_ (t)	i indicated student or employee	[3, 12]
County-level positive cases normalized by county population	c (t)		[13]
County-level positive cases normalized by county population (Age-categorized)	c_i_ (t)	i = student indicating normalized positive cases in 5–18 years old individuals; i = employee indicating normalized positive cases in individuals above 18 years old	[13]
Proportion of student and employee following the mask mandate in the community	${m}_{i}(t)$		[15]
In-person school days indicator	sd (t)	0 or 1	[10]
County-level seroprevalence	${r}_{i}(t)$	i = student indicating normalized positive cases in 5–18 years old individuals; i = employee indicating normalized positive cases in individuals above 18 years old	[14]

### Model parameters

In this study, transmission of SARS-CoV-2 was assumed to occur via two exposure routes: school and community. The in-school transmission of SARS-CoV-2 in unvaccinated or vaccinated individuals was modeled in equation [7.1] or [7.2], respectively.

7.1 $${\alpha }_{wave} \times \sum \frac{{\varnothing }_{k} \times {S}_{u,i} \left(t\right) \times \left({I}_{v,i} \left(t\right) + {I}_{u,i}\left(t\right)\right)}{\left(N - \sum {Q}_{i}\left(t\right)\right)}$$

7.2 $${\alpha }_{wave} \times \sum \frac{\left(1 - {e}_{i}\right)\times {\varnothing }_{k}\times {S}_{v,i}\left(t\right) \times \left({I}_{v,i}\left(t\right) + {I}_{u,i}\left(t\right)\right)}{\left(N - \sum {Q}_{i}\left(t\right)\right)}$$

Where ${\alpha }_{wave}$ is the estimated infectivity of the three waves of SARS-CoV-2 infections during the studied period from spring 2021 to spring 2022 [22]. ${S}_{u,j }(t)$, ${S}_{v,j }(t)$, ${I}_{u, j}(t)$, ${I}_{v, j}(t)$, and Q are the unvaccinated and vaccinated susceptible, vaccinated or unvaccinated infected, and quarantined population in the schools. N is the total school population; e is the efficacy of mRNA vaccine against infection in fully vaccinated students or employee population in the U.S. [20]; i indicates the role of the individuals in schools while j indicates the contacts between individuals. ${\Phi }_{k}$ is the contact and transmission coefficient between individuals (equation [8]).

8 $${\varnothing }_{k} = {\beta }_{k} \times sd\left(t\right)$$

Where ${\beta }_{k}$ is the product of daily contact and transmission probability based on contacts between individuals in schools; ${\delta }_{l}$ is the mask efficacy, when l represented the contact scenarios occurred between masked individuals (${\delta }_{mm}$) [19], a masked and an unmasked individual (${\delta }_{um}$), or two unmasked individuals (${\delta }_{uu}$); sd(t) is an indicator series representing days with (1) and without (0) in-person classes, assuming no in-person classes on weekends and holidays (0). The mask efficacy was estimated to be 0.44 between masked individuals and 1 between unmasked individuals. The mask efficacy between masked and unmasked individuals was used as one of the fitting parameters between ${\delta }_{mm}$ and ${\delta }_{uu}$. We assumed that in-school contacts were between students within their school year compared to those between students in different school years. Thus, ${\beta }_{k}$ represented the contact scenarios: 1) ${\beta }_{ss1}$, among students in primary schools (grade K-5), 2) ${\beta }_{ss2}$, among students in middle schools (grade 6 -8), 3) ${\beta }_{se1}$, between students and employees in primary schools (grade K-5), 4) ${\beta }_{se2}$, between students and employees in middle schools (grade 6 -8), 5) ${\beta }_{ee}$, between employees, and 6) ${\beta }_{se}$, between employee and students in all schools. ${\beta }_{k}$ except ${\beta }_{se}$ were obtained by the fitting process. We assumed limited interactions between primary and middle school students (${\beta }_{se}$ = 0) because of school district organization. We also assumed that the transmission rates between a student and an infected employee or an employee and an infected student was the same.

The community transmission of SARS-CoV-2 in susceptible students and employees was simulated by Equation [9]. In this equation, ${\varepsilon }_{j}$ was a factor simulating the contact and transmission rate between a susceptible student or employee with an infected community member and c(t) was the number of daily positive cases, normalized by the county-level population, in the county where the school was in.

9 $${\varepsilon }_{j} \times c\left(t\right)$$

The vaccination of students and employees over time was simulated by equation [10]. The daily increment in county-level vaccination rate, vr_i_(t), was calculated as the difference between the daily vaccination rates per county [3] in the studied period.

10 $${vr}_{i}\left(t\right) \times {S}_{u,i}\left(t\right)$$

Weekly testing results were reported by participating schools. The detection and quarantine of infected students and employees in the schools based on the weekly testing scheme were simulated by equation [11].

11 $${t}_{i}\left(t\right) \times \left({I}_{u,i}\left(t\right) + {I}_{v,i}\left(t\right)\right)$$

The recovery of the infected individual was simulated by multiplying the recovery rate by the infection and quarantine compartments. The recovery rate $\gamma$ was determined based on the reported infectious periods from three dominant variants of SARS-CoV-2. The rate of losing immunity in recovered individuals was simulated by $\omega$, which was obtained by the fitting process.

### ABC-SMC fitting process

An Approximate Bayesian Computation with sequential Monte Carlo (ABC-SMC) algorithm was used to estimate the unknown model parameters listed in Table 2. The priors of the model parameters were initialized by uniform distributions within the upper and lower bounds as specified in Table 2. The distance function between the simulated probabilities of infections and the incidence rate was calculated as in equation [12].

12 $$d = \sum_{t}^{T}{\left[{\sum_{i}\left[{t}_{i}\left(t\right) \times \left(P\left({I}_{u,i}\left(t\right)|{t}_{i}\left(t\right)\right)\right.+ P\left({I}_{v,i}\left(t\right)|{t}_{i}\left(t\right)\right)-P\left({Y}_{i}\left(t\right)\right)\right]}^{2}\right]}^{{}^{1\!}/_{\!\,2}}$$

In this equation, ${t}_{i}(t)$ were the daily number of students or employees who took the test after normalizing by the student or employee population, respectively. $P({I}_{u, i}(t)| {t}_{i}(t))$ and $P({I}_{v, i}(t)| {t}_{i}(t))$ were the simulated probabilities of infections in unvaccinated and vaccinated students or employees given the testing proportion in students or employees. $P({Y}_{i}(t))$ were the average daily incidence rates in students and employees, which were calculated by equation [13].

13 $$P\left({Y}_{i}(t)\right) = \frac{{n}_{i,m}(t)}{{N}_{school,m}} \times \frac{{N}_{school,m}}{\sum {N}_{school,m}}$$

In this equation ${n}_{i,m}(t)$ was the number of positive cases at day t in students or employees, as indicated by i, in school m; ${N}_{school, m}$ was the total population of the school m.

The ABC-SMC algorithm was used to produce 1000 parameter sets per fitting iteration. In the first iteration, all parameter sets were accepted. In each subsequent iteration, parameter sets were accepted if the distance (Equation [12]) was lower than the 75% percentile of the distance generated by the previous round of fitting, and the algorithm was repeated until 1000 parameter sets had been accepted. Ten iterations of initial fitting were carried out and followed by another 8 iterations of fitting.

### Model validation

To validate our model and the distributions of parameters obtained from the fitting process, we extended the simulated period from the middle of Spring 2022 (March 21, 2022) to the end of Spring 2022 (June 21, 2022) based on the parameters obtained from fitting process. The simulations were repeated 1000 times and the simulated incidence risks were compared to the reported infections normalized by the school populations (Equation [13]) from January 2021 to June 2022, which contained data that were withheld from the fitting process.

### Global sensitivity analysis

A global sensitivity analysis on the model parameters was conducted to assess the effects of the preventive measures demonstrated by parameters, such as internal and external transmission rates (${\beta }_{k}$) in schools and communities, the mask adherence (${m}_{i}$), and the testing proportions (${t}_{i}$), on SARS-CoV-2 infections using Latin Hypercube sampling (LHS). The posterior distributions of the within-school and external transmission rates (${\beta }_{ss1}$, ${\beta }_{ss2}$, ${\beta }_{se1}$, ${\beta }_{se2}$, ${\beta }_{ee}$, ${\varepsilon }_{s1}$, ${\varepsilon }_{s2}$, and ${\varepsilon }_{e}$ as shown in Table 3) and two uniform distributions of mask adherence and testing proportion from 0 to 1 were divided into 200 equiprobable serial intervals. One hundred parameter sets were constructed by randomly drawing values from the equiprobable serial intervals and used in the sensitivity analysis. Distributions of the other model parameters except for the aforementioned derived parameters remained the same as shown in Tables 2 and 3. For each set of parameters, 100 simulations were conducted based on the values drawn from other parameters, including the infectiousness of the predominant variants in the three waves, efficiencies of vaccines and masks, and loss of immunity, which were randomly drawn from the empirical distributions obtained from the fitting process. One of the weekdays was randomly selected as the testing day over the simulated periods. The global sensitivity analysis was carried out in school demographics categorized based on the 10, 50, and 90% percentiles in total school populations, which was reported in the ElSi database [12]. The correlations between the model parameters and the simulated incidence risks were demonstrated by partial rank correlation coefficients (PRCC). The sign of the PRCC represent the positive or negative correlation and the magnitude of the PRCC show the level of correlation between the model parameters and the simulated incidence risks.

**Table 3:** derived model parameters obtained from the ABC-SMC fitting process corresponding

Derived model parameters	Parameter	Medians	95% confidence interval
Transmission among students in kindergarten to 5th grade	${\beta }_{ss1}$	0.064	[0, 0.38]
Transmission among students in 6th to 8th grade	${\beta }_{ss2}$	0.12	[0, 0.59]
Transmission between employee and students in kindergarten to 5th grade	${\beta }_{se1}$	0.010	[0, 0.15]
Transmission between employee and students in 6th to 8th grade	${\beta }_{se2}$	0.018	[0, 0.31]
Transmission among employees	${\beta }_{ee}$	0.65	[0.028, 1.1]
Loss of immunity rate	$\omega$	0.79	[0.32, 1.0]
Transmission between masked and unmasked individual	${\delta }_{mu}$	0.77	[0.49, 0.96]
External transmission to students in kindergarten to 5th grade	${\varepsilon }_{s1}$	0.78	[0, 1.6]
External transmission to students in 6th to 8th grade	${\varepsilon }_{s2}$	1.1	[0, 3.0]
External transmission to employee	${\varepsilon }_{e}$	0.90	[0, 2.8]
Infectivity of first wave	${\alpha }_{1}$	1.1	[1, 1.3]
Infectivity of second wave	${\alpha }_{2}$	1.0	[1, 1.2]
Infectivity of third wave	${\alpha }_{3}$	1.5	[1, 2.4]

### Scenario analysis on interventions of the testing programs

We also simulated the incidence risks under different testing programs instead of weekly testing. First, we considered increasing the frequency of testing from weekly to every weekday under various testing proportions. In this scenario, four testing proportions (0, 25%, 50%, 100%) in students and employees were simulated in each testing. The testing day was randomly selected on a weekday for students and employees, respectively, for the weekly testing condition. Next, we considered trigger testing, in which all students and employees were subjected to testing when either 1) ten or more infections or 2) ten or more percent of the school population were found in the school districts. Each scenario was simulated in 1000 iterations by randomly drawing from the distributions of parameters obtained during the fitting process. The outputs of these scenario analyses were compared to the observed and simulated values under the weekly testing program implemented in the studied school districts.

### Statistical analysis

All simulations were conducted in R version 4.2.2. Significance was determined for *p* < 0.05 obtained from each hypothesis tests. Data cleaning and reorganization were carried out with package “dplyr” [23], “tidyverse” [24], “reshape2” [25], “stringdist” [26], “lubridate” [27], “MMWRweek” [28] and “fuzzyjoin” [29]. The global sensitivity analysis was carried out with package “lhs” [30] and “epiR” [31]. Figures were generated by package “ggplot2” [32].

## Results

County-level vaccination and simulation of weekly infections in school districts.

**Fig. 3:**
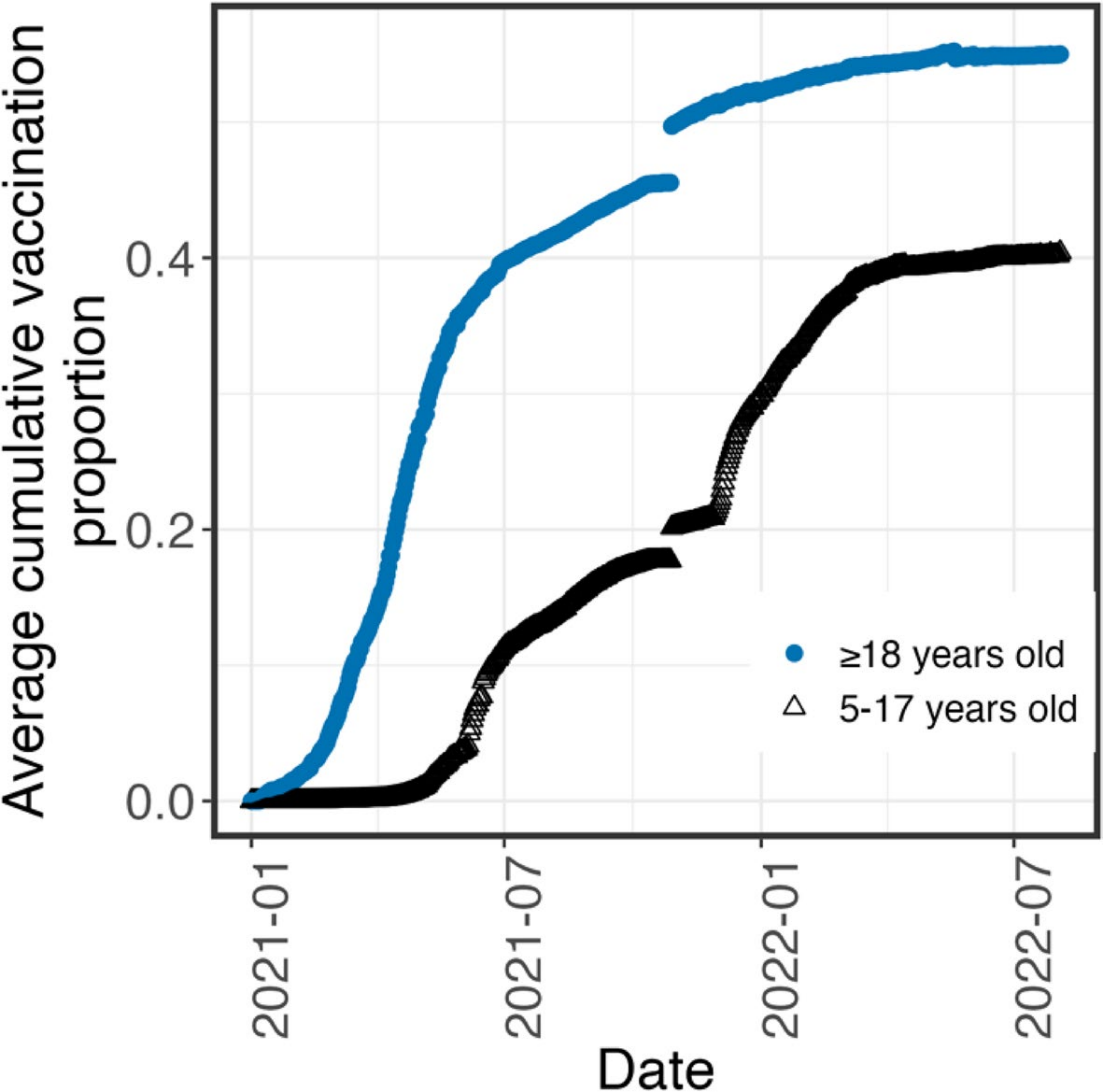
The average cumulative vaccination proportions of individuals in the respective age ranges reported to be vaccinated with a two-doses primary series in Illinois in two age groups: from 5–17 years old (black opened triangle) and equal to or greater than 18 years old (blue solid circle)

**Fig. 4:**
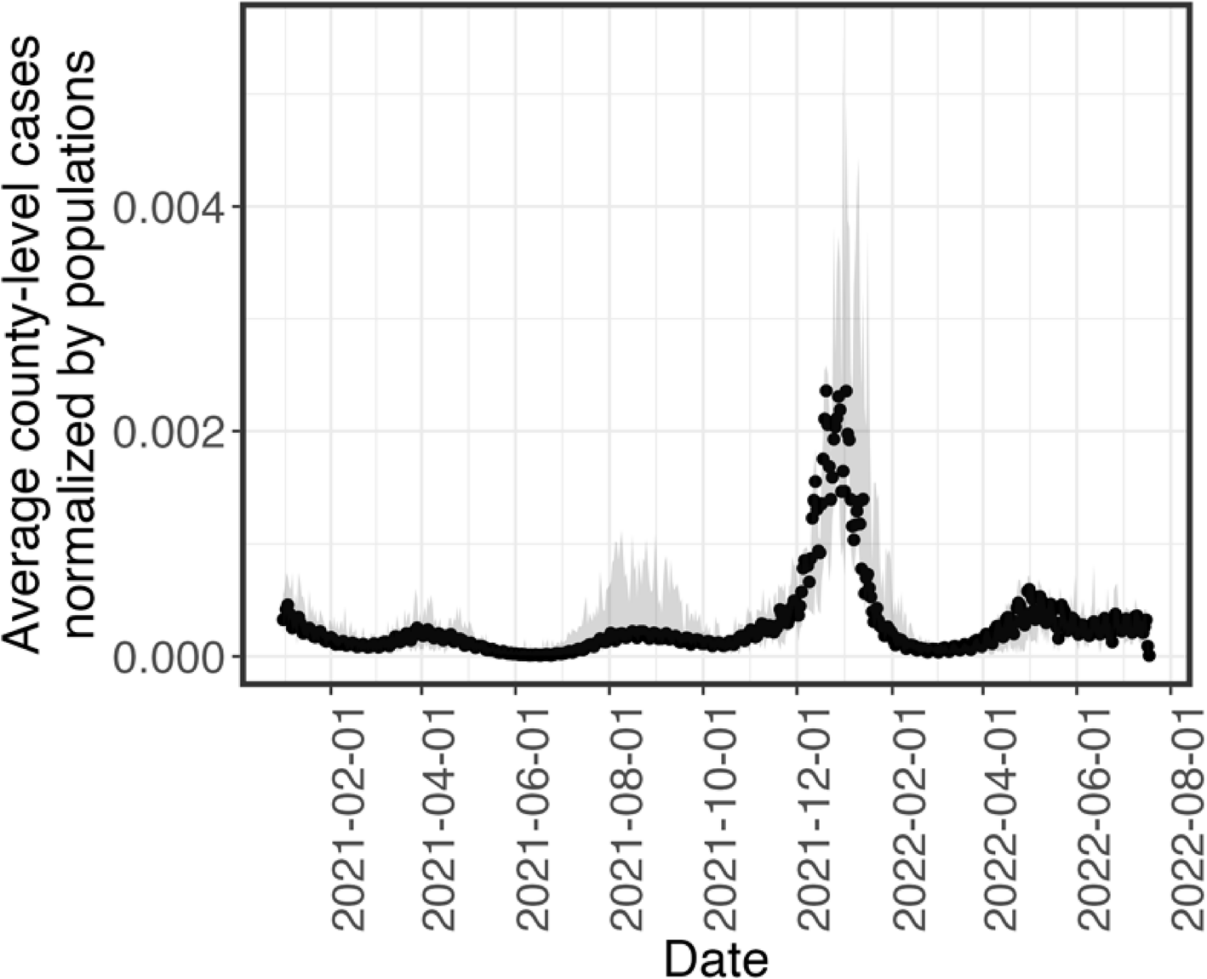
The average daily number of county-level positive cases normalized by the county-level population in Illinois (solid circle) during the studied period (January 2021 to July 2022). The shaded area is the daily minimum and maximum of the normalized positive cases

We used testing results obtained from 116 public school districts with primary and middle schools in Illinois, USA, from January 2021 to June 2022. As shown in Fig. 1, the majority of the participating school districts were in Cook (52%), Lake (13%), and Dupage (8.5%) Counties, which have the highest population of counties in the state. Most of the school districts were in urban and suburban locales (82%), while the rest of the school districts’ locations were considered small towns or rural. According to the 2020–2022 U.S. Census, the median population size of the communities associated with the studied school districts was 1.4 $\times$ 10^5^ (95% CI: 2.9 $\times$ 10^4^—2.4 $\times$ 10^6^). The average vaccination proportions in students and employees in the primary and middle school districts were approximated by the county-level vaccination rates reported by CDC [3], as shown in Fig. 3. The vaccination proportion was determined based on the number of individuals completing a two-dose primary series of mRNA vaccines after normalization by the county-level populations. Due to the early vaccination availability to adult in 2021, the average vaccination proportion in adults started to increase in the beginning of Spring 2021 while the average vaccination proportion in youths from 5 to 17 years old started to increase by the end of Spring 2021 (Fig. 3). The average positive rate was obtained by the daily positive cases reported by counties and normalized by the county-level population as shown in Fig. 4. Three waves of infections were observed from Spring 2021 to Spring 2022 in the communities where the studied school districts resided.

### Simulations of SARS-CoV-2 transmission in students and employees in primary and middle school districts

**Fig. 5:**
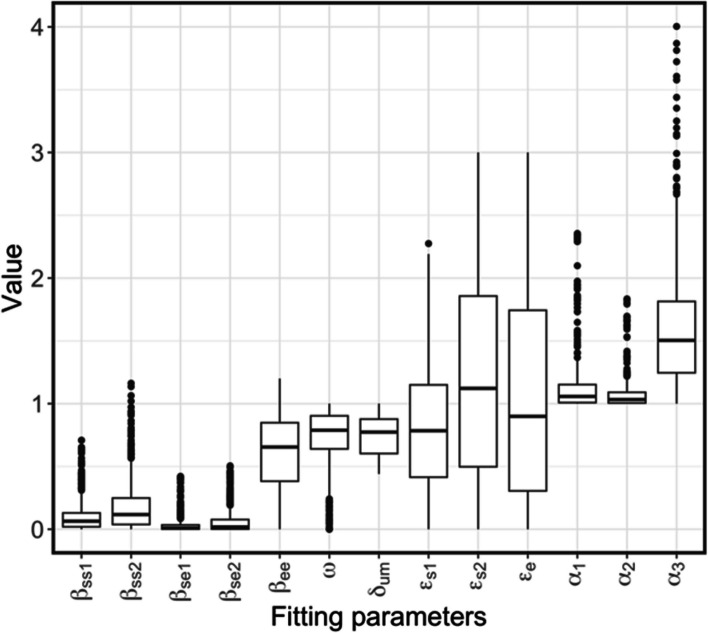
The distributions of fitted parameters for the model of SARS-CoV-2 transmission in school districts consisting of primary and middle schools. The solid lines are medians, and the boxes represent the 25–75% percentile of the parameters. The whiskers are 1.5 times the internal quantile range (IQR) and the dots are the outliers

The distributions of parameters obtained from the fitting process are shown in Fig. 5 and Table 3. For within-school transmission rates, the transmission rates among employees (${\beta }_{ee}$, medians: 0.65, 95% CI: 0.028–1.1) were significantly higher, followed by those among middle school students (${\beta }_{ss2}$, medians: 0.12, 95% CI: 0–0.59) and those among primary school students (${\beta }_{ss1}$, medians: 0.064, 95% CI: 0–0.38). The transmission rates between employees and primary (${\beta }_{se1}$, medians: 0.010, 95% CI: 0–0.15) or middle school students (${\beta }_{se2}$, medians: 0.018, 95% CI: 0–0.31) were the lowest. As shown in Fig. 5 and Table 3, the estimated external transmission rates in all population groups (${\varepsilon }_{s1}$, ${\varepsilon }_{s2}$, ${\varepsilon }_{e}$) were significantly greater than the estimated within-school transmission rates (${\beta }_{ss1}$, ${\beta }_{ss2}$, ${\beta }_{se1}$, ${\beta }_{se2}$, ${\beta }_{ee}$). The estimated external transmission rates among middle school students were statistically greater than those among employees and followed by those among primary school students. The infectivity of the second wave were significantly the lowest (Fall 2021, ${\alpha }_{2}$, medians: 1.0, 95% CI: 1–1.2), slightly followed by the infectivity of the first wave (Spring 2021, ${\alpha }_{1}$, medians: 1.1, 95% CI: 1–1.3); the infectivity of the third wave (Spring 2022, ${\alpha }_{3}$, medians: 1.5, 95% CI: 1.-2.4)) was the highest. This increased infectivity of the third wave compared to the first wave has been previously reported in [33].

**Fig. 6:**
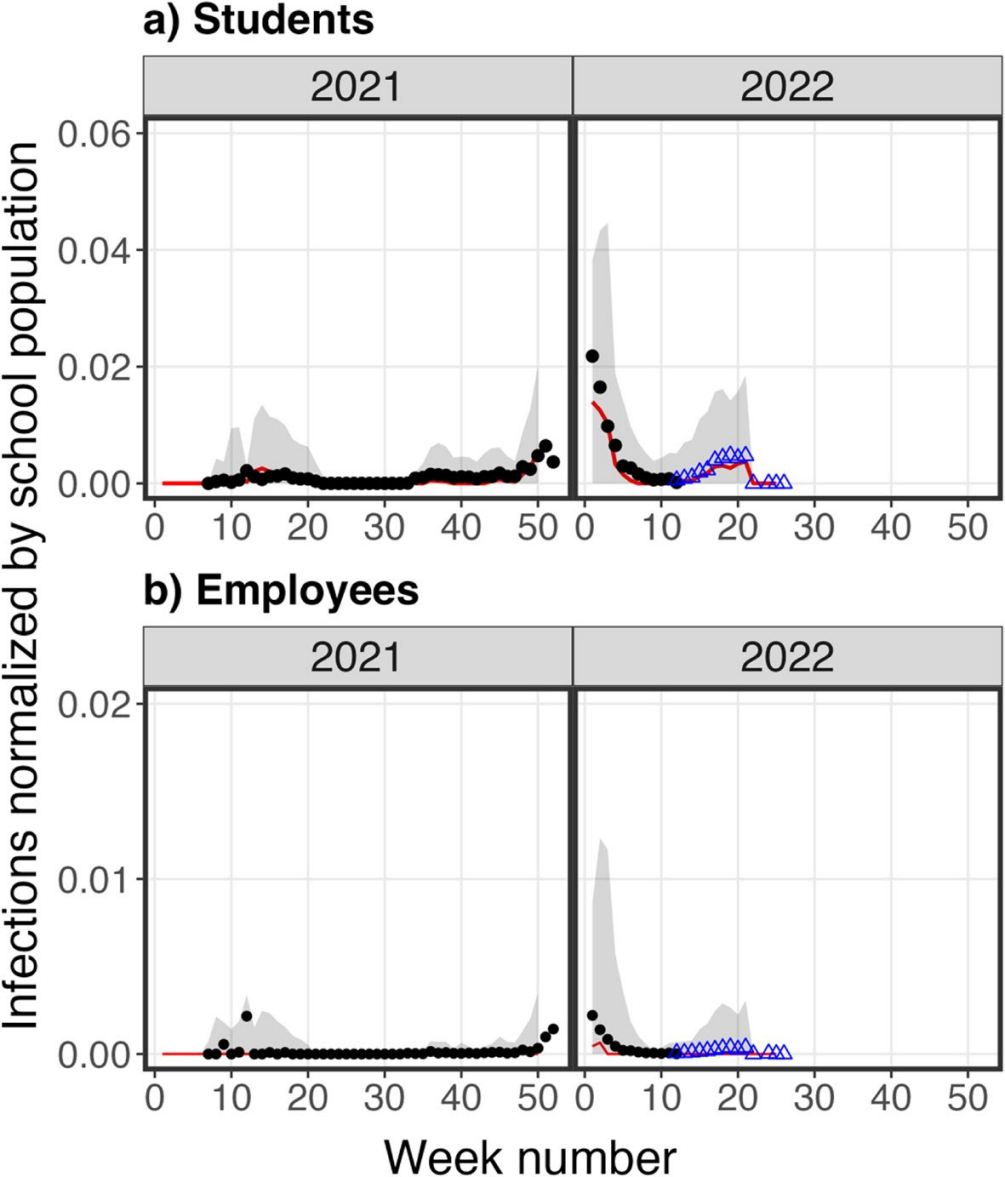
The reported (solid circles and opened triangles) and simulated (solid red lines and shaded area) weekly-averaged incidence risks in **a**) students and **b**) employees of primary and middle school districts from Spring 2021 to Spring 2022. The black solid circles are the reported average incidence risks used in the model fitting process. The blue opened triangles are the reported average incidence risks used in the model verification process. The red lines are medians of the simulated weekly incidences and the shaded areas represent 95% prediction intervals

As shown in Fig. 6, the simulated incidences in students and employees from January 2021 to March 2022 agreed with the reported infections in students and employees in the primary and middle school districts from the same period. Also, the model and the fitting parameters obtained based on the surveillance testing from January 2021 to March 2022 was able to predict the observed infection incidence from March 2022 to June 2022, which was withheld from the fitting process (Fig. 6).

### Global sensitivity analysis

**Fig. 7:**
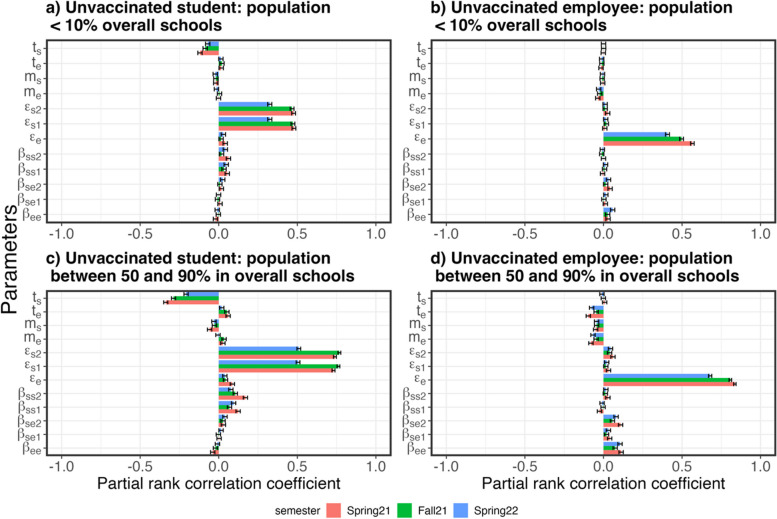
Representative PRCCs of model parameters from a global sensitivity analysis on SARS-CoV-2 incidence risks in unvaccinated students (**a** and **c**) and employees (**b** and **d**) in the studied primary and middle school districts with populations below 10% (**a** and **b**) and between 50 and 90% (**c** and **d**) of the overall schools population. Solid bars show medians of PRCCs in Spring 2021 (pink), Fall 2021 (Green), and Spring 2022 (Blue). Error bars show the 95% confidence interval

Figure 7 and Table S1 show the representative linear correlations between the model parameters and the simulated infection rates as estimated by PRCC from a global sensitivity analysis. PRCCs of model parameters in vaccinated and unvaccinated students and employees in four school districts sizes are shown in Figure S1 to S4. Overall, the most important factors in incidence risks in students were external transmission rates and testing proportions in students in primary and middle schools. Similarly, the most important factors in incidence risks in employees were external transmission rates and testing proportions in employees. The global sensitivity analysis showed that lower PRCCs were found for all within-school transmission rates compared to external transmission rates, which agreed with the parameters obtained in the fitting process (Fig. 5). The external transmission rates in the middle school students were not significantly driving the incidence risks in employees when the school population was small (school districts with populations under 10% of populations in all participating school districts). The PRCCs for the within-school transmission rates between students and employees in both middle schools and primary schools increased incidence risks in employees in larger school districts (those with populations greater than 10% of populations in participating school districts). Also, since the proportions of vaccinated students and employees increased during the studied period, the PRCC of all parameters decreased over time from Spring 2021 to Spring 2022 in the unvaccinated population and increased in the vaccinated population (Figure S1-S4). As shown in Fig. 7, the PRCCs of within-school transmission rates increased with increasing school populations. The PRCCs of transmission rates in middle school students were greater than those in primary school students. The PRCCs of transmission rates between students and employees were greater than those among employees.

### Scenario analysis on testing frequency in primary and middle schools

**Fig. 8:**
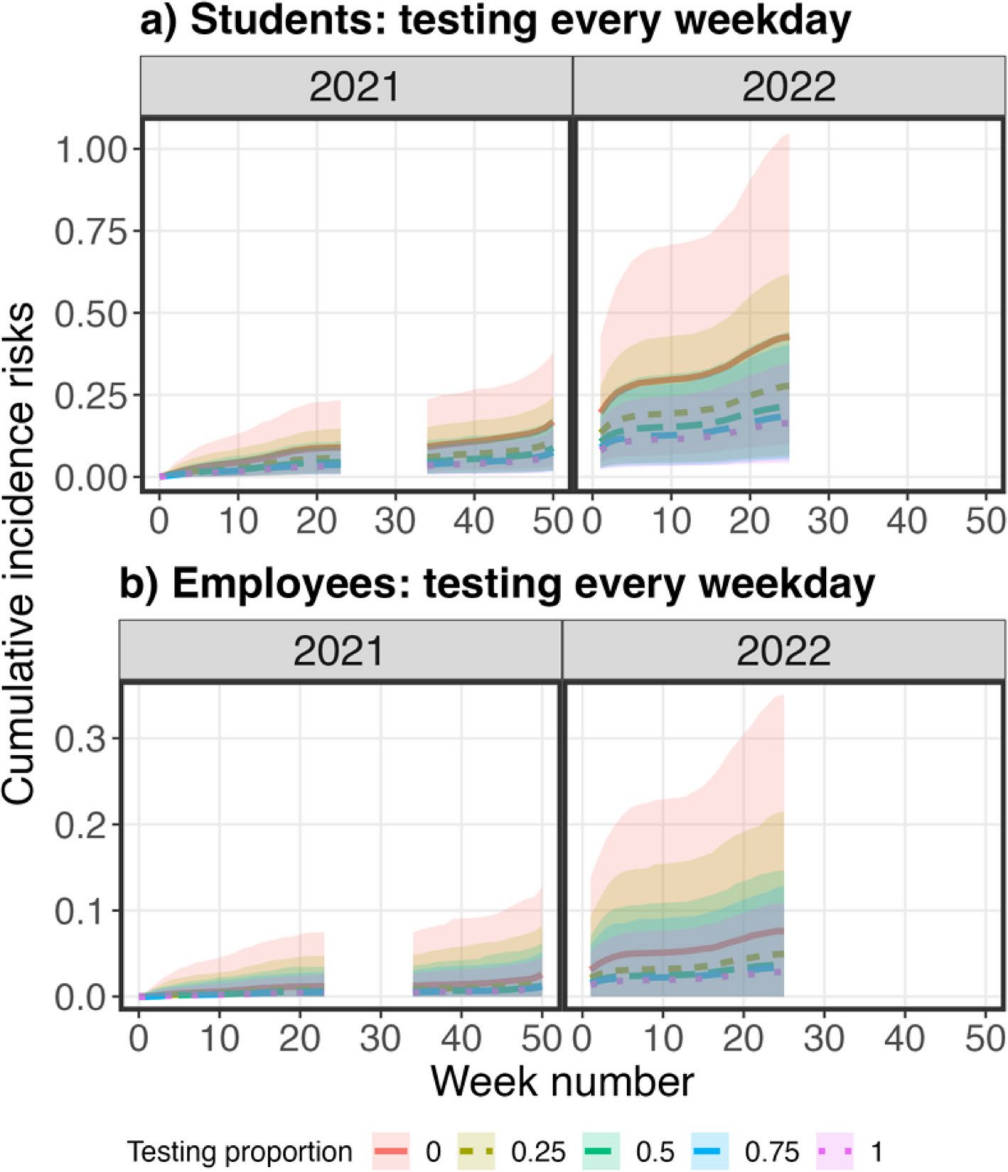
The cumulative predicted incidence risks in students and employees when the testing proportions in students (**a**) and employees (**b**) were 0 (red), 25% (green), 50% (blue), 75% (blue), and 100% (purple). The testing frequency is every weekday. The solid lines were the medians of the cumulative predicted incidence risk and the shaded areas were the 95% confidence intervals

**Fig. 9:**
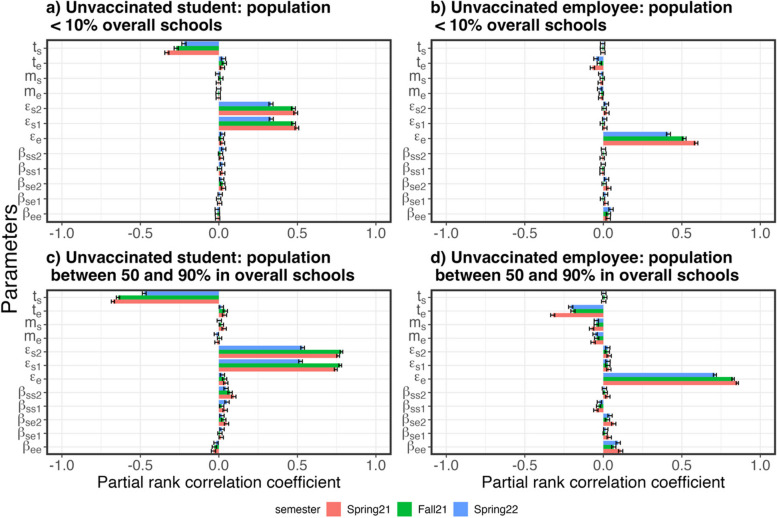
Representative PRCCs of the model parameters on incidence risks under the testing frequency of each weekday per week in unvaccinated students (**a** and **c**) and employees (**b** and **d**) in school districts with population under 10% (**a** and **b**) and between 50 to 90% (**c** and **d**) of total population. The bars are the medians and the error bars are 95% confidence intervals

Based on the scenario analysis conducted on two testing frequencies (once every week and every weekday), the predicted incidence risks in students and employees decreased with increased testing proportions in the students and employees (Figure S5 and Fig. 8). When the testing frequency increased from once per week to each weekday, the reductions in the predicted incidence risks increased in both students and employees (Figure S5 and Fig. 8). These changes in the reductions of incidence risks with increased testing frequency were also reflected in a global sensitivity analysis with the assumption of testing every weekday (Fig. 9, Table S2 and Figure S6 to S9). Similar to the lower testing frequency scenario, external transmission rates and testing proportions in students and employees were ranked as the most significant parameters in the incidence risks. However, the PRCCs of testing proportions in students and employees increased when the testing frequency increased from once per week to each weekday per week. These changes in PRCCs suggested that the selection of testing proportions, especially in students, can reduce infections when testing is implemented frequently.

### Scenario analysis on interventions in school testing program

**Fig. 10:**
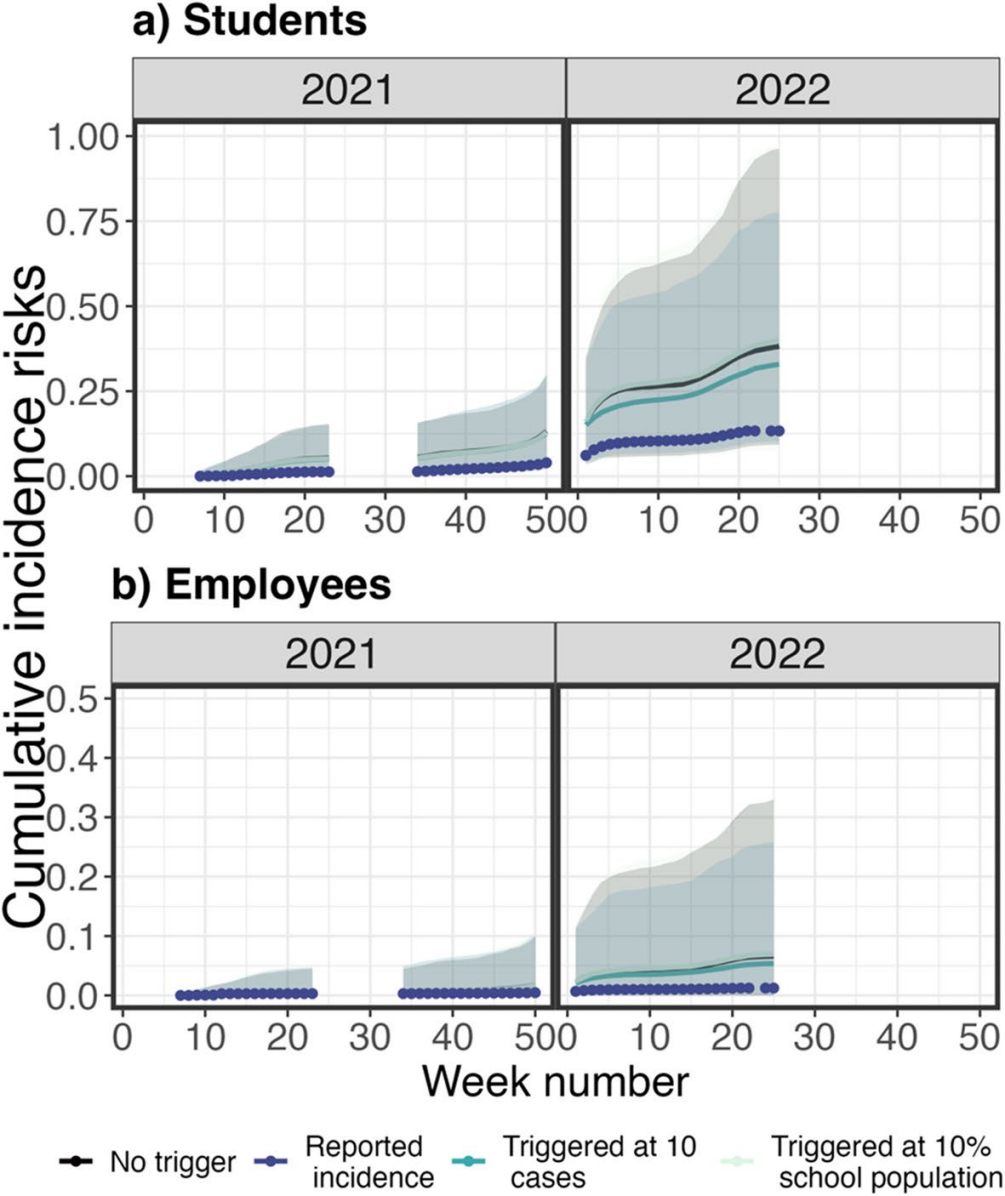
Cumulative incidence risks of school districts operated under a trigger-testing and quarantine program in students (**a**) and employees (**b**). The following trigger scenarios were considered: No trigger (Dark purple), 10 infections associated with school (Green), and 10% of school population was infected (Light green). The solid lines are medians of the cumulative incidence risks, and the color bands are 95% confidence interval. The solid points are observed cumulative incidence risks (Light purple). The gaps between data are summer and winter breaks in 2021 and 2022

We also simulated the incidence risks of students and employees under a trigger-testing program, in which testing would be implemented after schools reached 10 cases or 10% of school population. As shown in Fig. 10, the medians of cumulative incidence risks of school districts under any trigger-testing and quarantine programs were greater than the reported infections. Among all trigger-testing and quarantine programs, the medians of cumulative incidence risks were lowest when all students and employees were tested if ten or more infections were found in the school districts. The differences between the medians of cumulative incidence risks in a trigger-testing program with ten or more infections and those in a no-testing school district were greatest in Spring 2022 when the distribution of infectiousness of the third wave was higher than in the first and second wave. These results of simulated incidence risks showed that the trigger-testing program could lead to more infections than the weekly testing program, but fewer infections than no testing.

## Discussion and limitations

In this study, we found that community transmissions of SARS-CoV-2 contributed to the incidence risks in students and employees in primary and middle school districts with indoor mask mandates and weekly testing programs implemented during the period of hybrid and in-person instruction in the Spring 2021 to Spring 2022. In our study, although the reported and simulated incidences in employees were lower than those in students, the estimated transmission rates among school employees were significantly greater than those among primary or middle school students. This result agreed with the observations reported in a review that found greater infection rates in school staff than in students [34]. Specifically, our estimations on greater secondary transmission risks in middle school students than those in primary school students also agreed with the results reported in an evaluation of test-to-stay programs in 90 schools in Lake County, Illinois, in Fall 2021 [35]. Also, our simulations showed that the within-school transmission rates were lower than the external transmission rates from the community in students and employees. These results agreed with the observations of modest secondary transmission in K-12 schools under a mask-on-mask or universal mask policy implemented either by governments or school districts in studies conducted in a similar timeframe to our studied period [8, 36–41]. The correlations between community incidences and school-related infections were reported in districts and charter schools in Florida [42], North Carolina and Wisconsin [8, 43]. Our primary finding, that community-acquired infections drive school cases, is consistent with the literature. This study found a strong link between the community incidence and school-affiliated incidence, as had been previously reported in an evaluation of school reopening in Indiana in Fall 2020 [44]. We found that there were likely to be 1.4 to 12.2 community-acquired infections per within-school transmission across the study period, which is lower than the 20-to-1 ratio observed in a large diverse cohort in Spring 2021 [8]. The shorter state-level mask mandate duration in the cohort may lead to the discrepancy of the ratio between the community-acquired infection and within-school transmission. Greater external transmission of SARS-CoV-2 compared to in-school transmission was also reported in elementary and middle schools in Japan [45] and Belgium [46]. Our results also showed that the testing proportions and frequencies can significantly reduce SARS-CoV-2 transmissions in schools, which was consistent with the previous studies [39, 47, 48].

However, this study has several limitations. First, we assumed the adherence to mask mandates in students and employees remained was consistent within and across schools, as we lacked data on the commitment to mask-wearing behaviors for students outside of schools. This assumption may reduce the simulated impact of mask adherence on incidences. However, the importance of this assumption may be limited because state-wide mask mandates for public spaces were in place throughout the studied period, and studies have shown that the willingness to wear a mask in public increased during the implementation of the mask mandate [49]. However, the conclusions on the correlation between school mask mandates and SARS-CoV-2 cases can be complicated in the U.S. due to the heterogeneity in the operational conditions and socioeconomic factors in counties and school districts [50]. In this study, we also found that self-reported local adult mask adherence was not highly ranked in the important factors for incidence risks in students and employees during the studied period, when a state-wide mask mandate was in place within schools and public spaces.

Another important assumption in this study was that participation of the school districts in the weekly testing program was consistent across the state during the studied period. The observed weekly-aggregated infections could be driven by school districts that with higher participation in testing, which might also be more likely to enforce social distancing and disinfection [48]. Previous studies have shown the tendency of parents or guardians to consent to test or vaccinate their children against SARS-CoV-2 can depend on multiple factors including test accessibility, family resources and impact, education, vaccination status, and SARS-CoV-2 vaccine hesitancy [51–53]. The schools in this study were highly heterogeneous in the socioeconomic status of populations served, which may limit our ability to draw broad conclusions. Future research will have to consider the role of individual school factors, such as location and student demographics, in model fitting and prediction accuracy.

## Conclusions

Based on weekly-testing results obtained from primary and middle public school districts across Illinois, we built a compartment model that simulated the incidences risks of SARS-CoV-2 in students and employees in those school districts across the time period of Spring 2021 to Spring 2022, which covered three waves of infections in the community. We found that the community transmission rates were greater than within-school transmission rates in students and employees. Simulations indicated that testing proportions and frequency can significantly reduce the incidence risks in students and employees. However, increased economic and social cost can be associated with the increased testing coverage and frequency. To aid in the policy making to prevent transmission in elementary and middle schools, further studies on the cost-effectiveness of the protective measures on reduction of infections are important.

## Supplementary Information

Supplementary Material 1.

## Data Availability

The datasets used and/or analyzed during the current study are available in the Illinois Data Bank (10.13012/B2IDB-3705306_V1). The code of the SIR model is available in GitHub (https://github.com/chuang3415/SHILED_K8_SIR.git).

## Abbreviation

SARS-CoV-2: Severe acute respiratory syndrome coronavirus 2
