# Supplementary material for: A modeling study on SARS-CoV-2 transmissions in primary and middle schools in Illinois
Source: BMC Public Health. 2024 Nov 18;24:3197. doi: 10.1186/s12889-024-20623-5 (PMC11572532; doi:10.1186/s12889-024-20623-5)
Supplement: Supplementary file 1 — Supplementary Material 1. [file 12889_2024_20623_MOESM1_ESM.docx]

# A modeling study on SARS-CoV-2 transmissions in primary and middle schools in Illinois

Supplemental Information

Conghui Huang^1^, Rebecca Lee Smith^1, 2, 3^

1. Department of Pathobiology, College of Veterinary Medicine, University of Illinois at Urbana Champaign, Urbana, IL, USA
2. Carle-Illinois College of Medicine, University of Illinois at Urbana Champaign, Urbana, IL, USA
3. Institute of Genomic Biology, University of Illinois at Urbana Champaign, Urbana, IL, USA


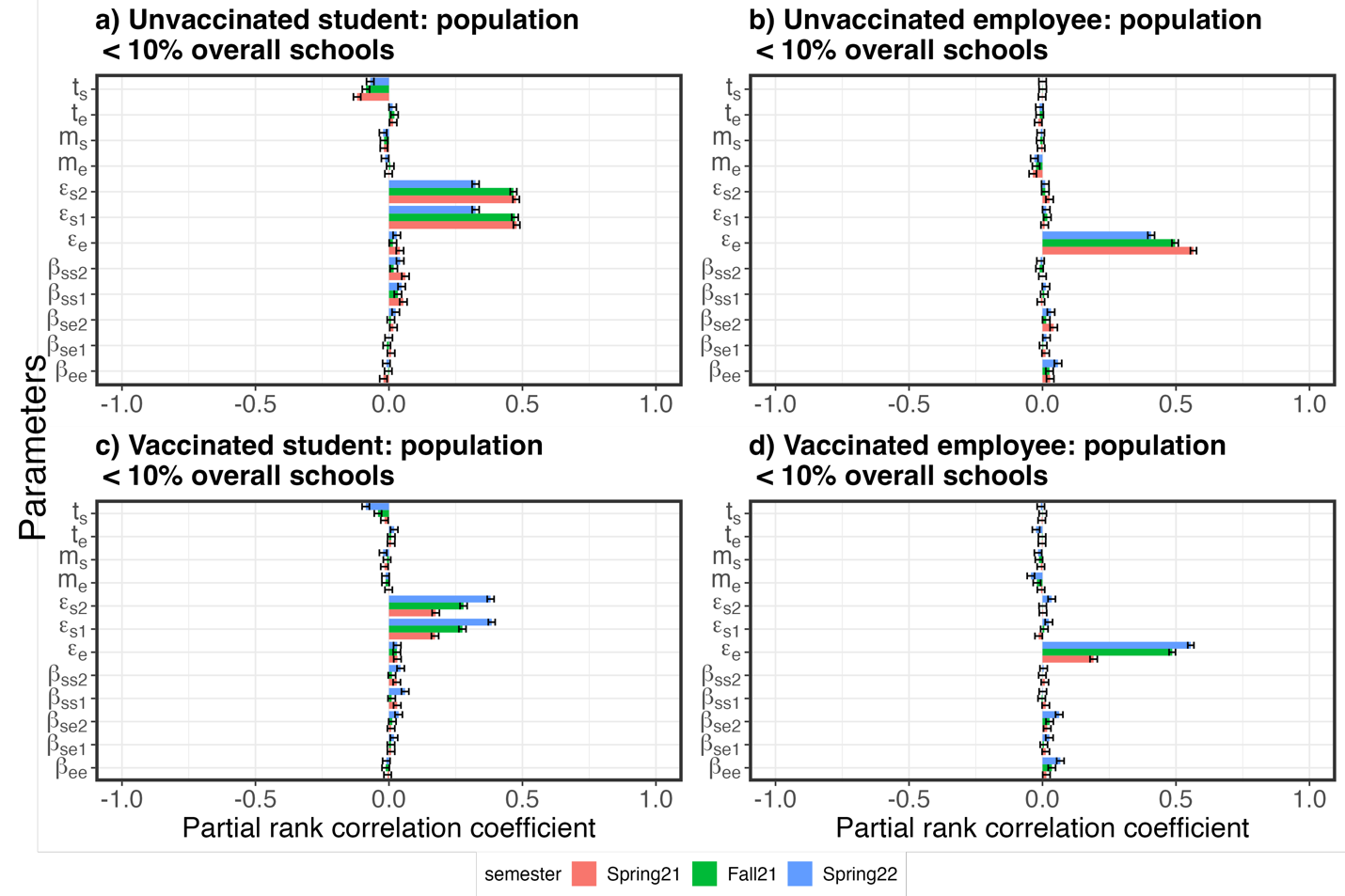


**Figure S1**, PRCCs of model parameters from a global sensitivity analysis on SARS-CoV-2 incidence risks in unvaccinated and vaccinated students (a and c) and employees (b and d) in the studied primary and middle school districts below 10% of the overall school population. Solid bars show medians of PRCCs in Spring 2021 (pink), Fall 2021 (Green), and Spring 2022 (Blue). Error bars show the 95% confidence interval.


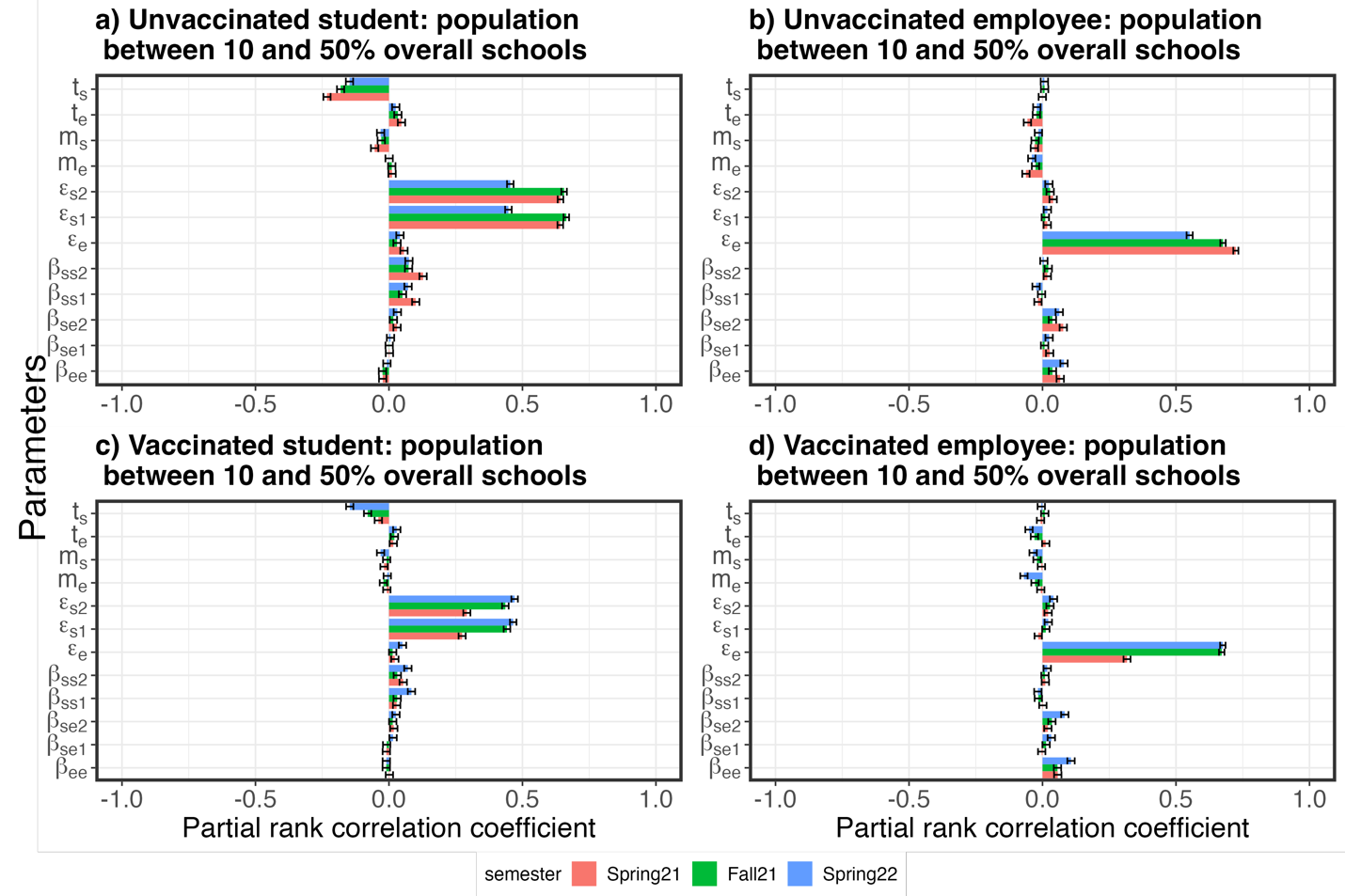


**Figure S2**, PRCCs of model parameters from a global sensitivity analysis on SARS-CoV-2 incidence risks in unvaccinated and vaccinated students (a and c) and employees (b and d) in the studied primary and middle school districts between 10% and 50% of the overall school population. Solid bars show medians of PRCCs in Spring 2021 (pink), Fall 2021 (Green), and Spring 2022 (Blue). Error bars show the 95% confidence interval.


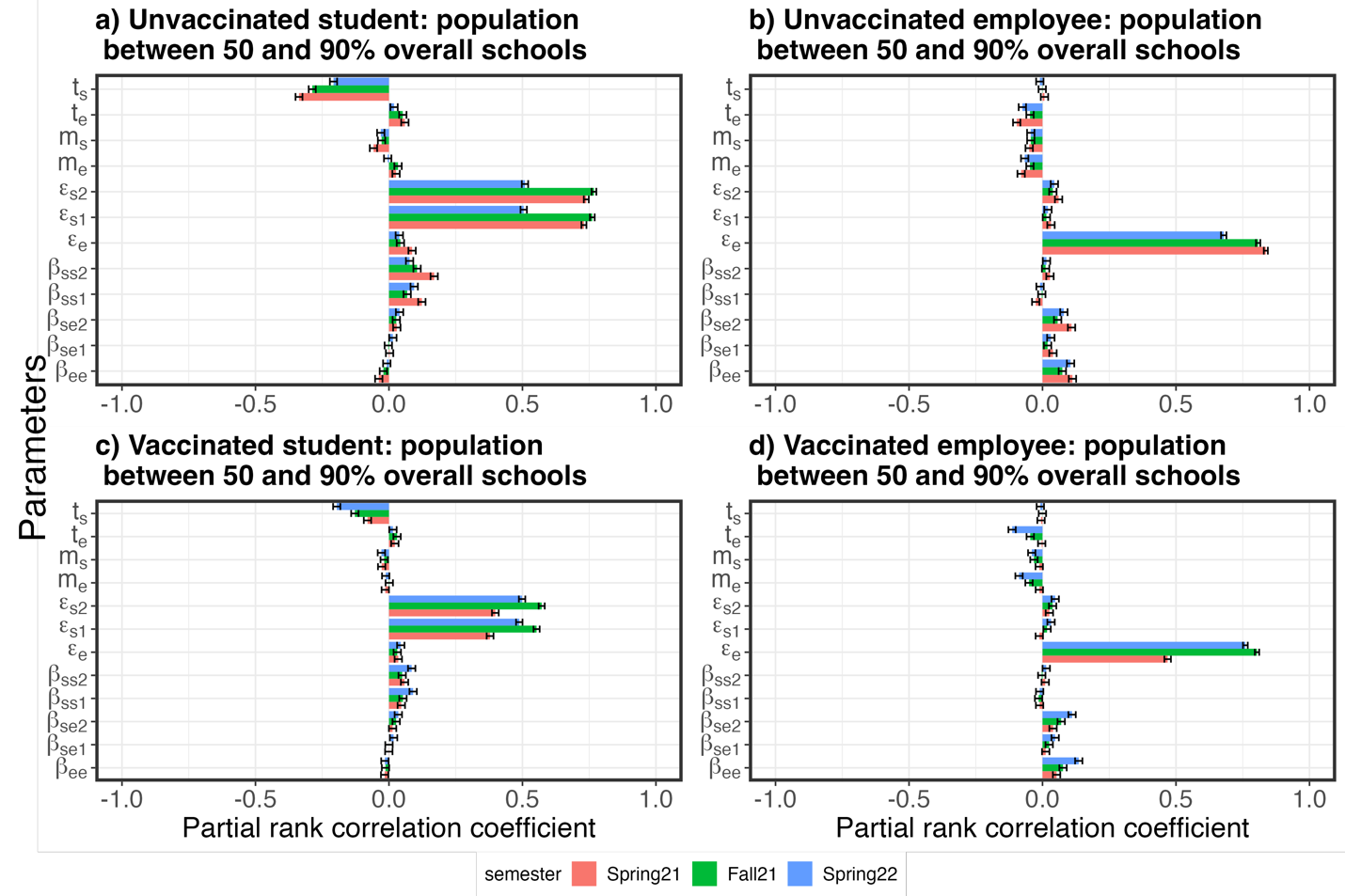


**Figure S3**, PRCCs of model parameters from a global sensitivity analysis on SARS-CoV-2 incidence risks in unvaccinated and vaccinated students (a and c) and employees (b and d) in the studied primary and middle school districts between 50% and 90% of the overall school population. Solid bars show medians of PRCCs in Spring 2021 (pink), Fall 2021 (Green), and Spring 2022 (Blue). Error bars show the 95% confidence interval.


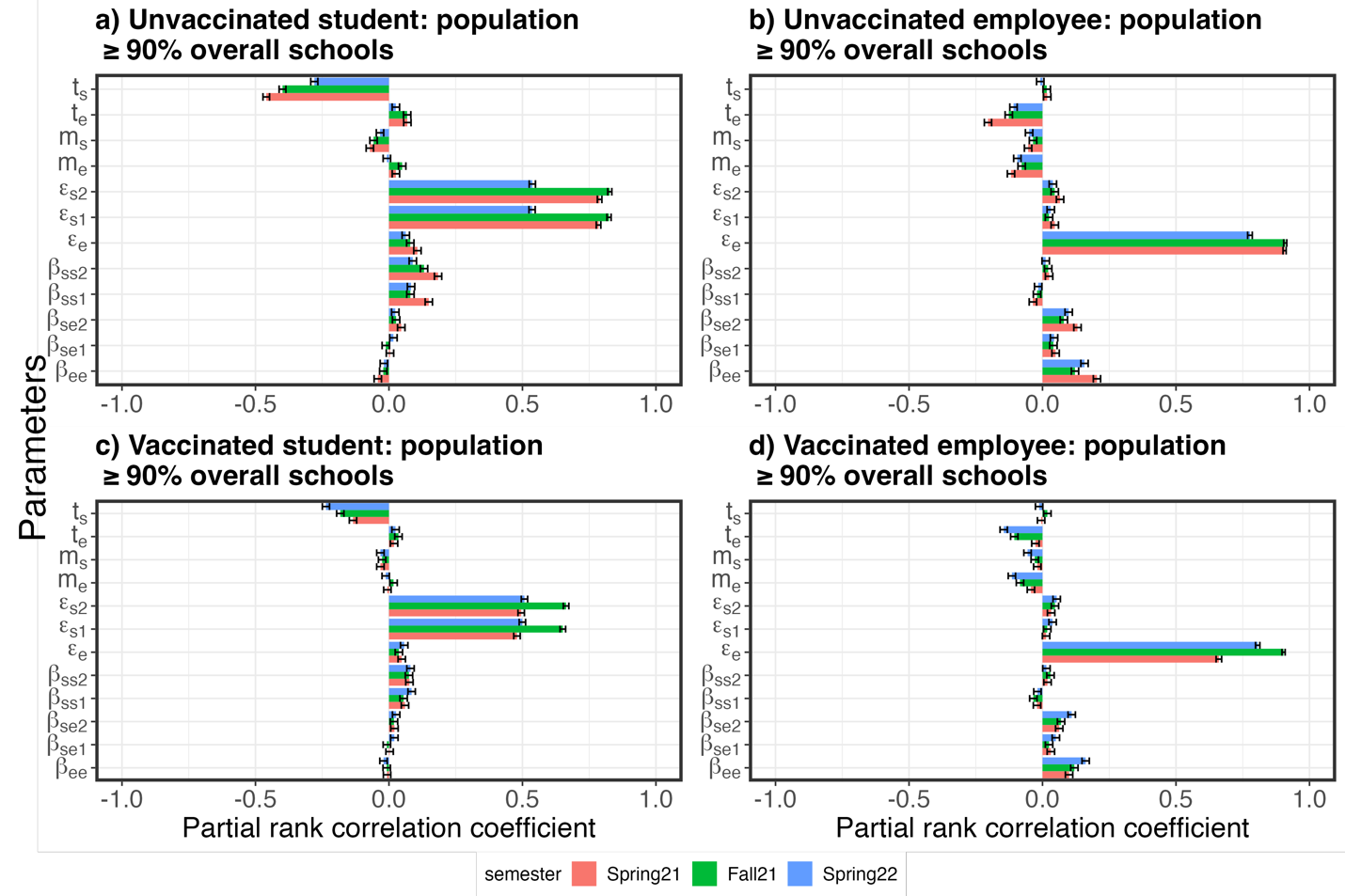


**Figure S4**, PRCCs of model parameters from a global sensitivity analysis on SARS-CoV-2 incidence risks in unvaccinated and vaccinated students (a and c) and employees (b and d) in the studied primary and middle school districts over 90% of the overall school population. Solid bars show medians of PRCCs in Spring 2021 (pink), Fall 2021 (Green), and Spring 2022 (Blue). Error bars show the 95% confidence interval.


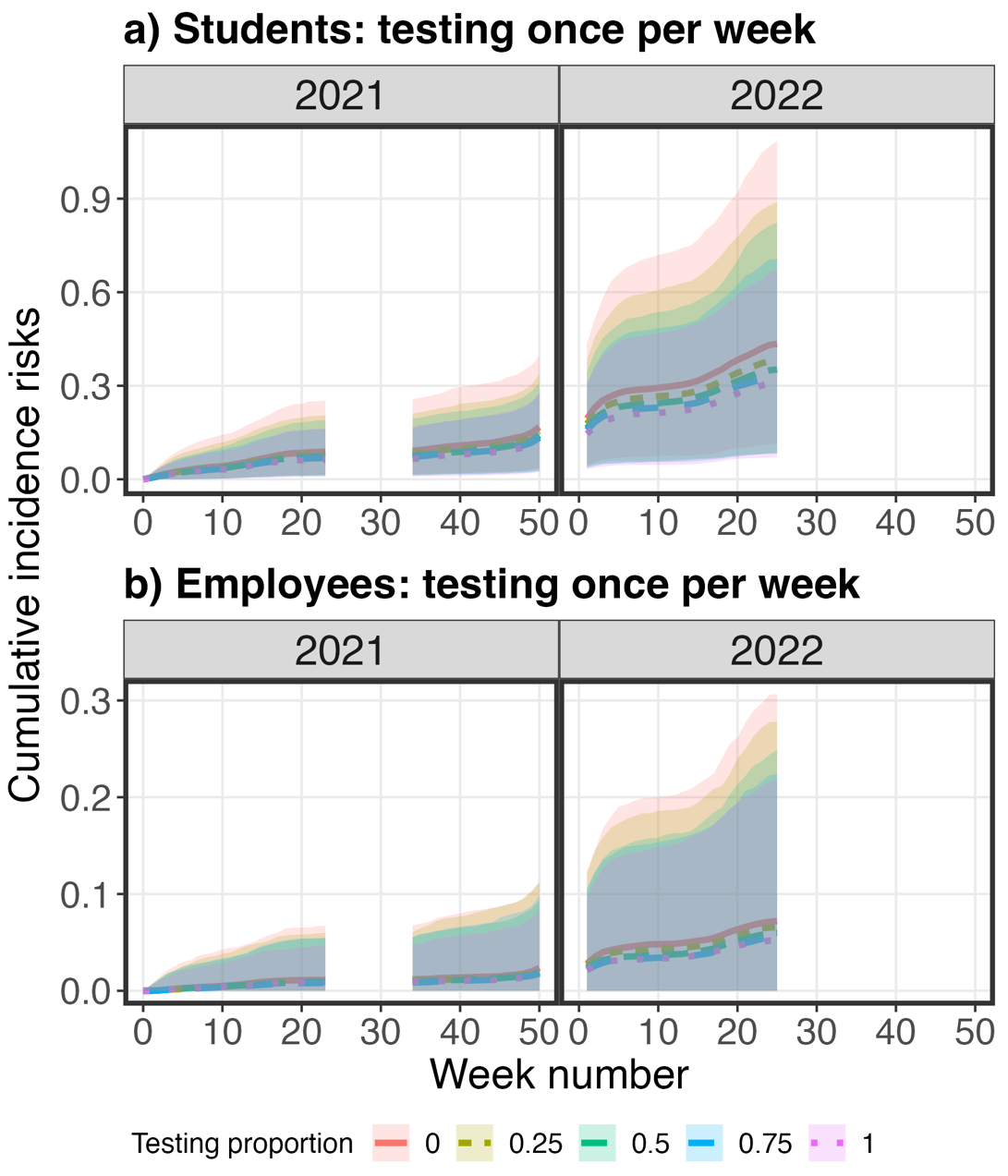


**Figure S5**, the cumulative predicted incidence risks in students and employees when the testing proportions in students (a and c) and employees (b and d) were 0 (red), 25% (green), 50% (blue), 75% (blue), and 100% (purple). Panel a) and b) show testing frequency at once per week while c) and d) show testing frequency at every weekday. The solid lines were the medians of the cumulative predicted incidence risks and the shaded areas were the 95% confidence intervals.


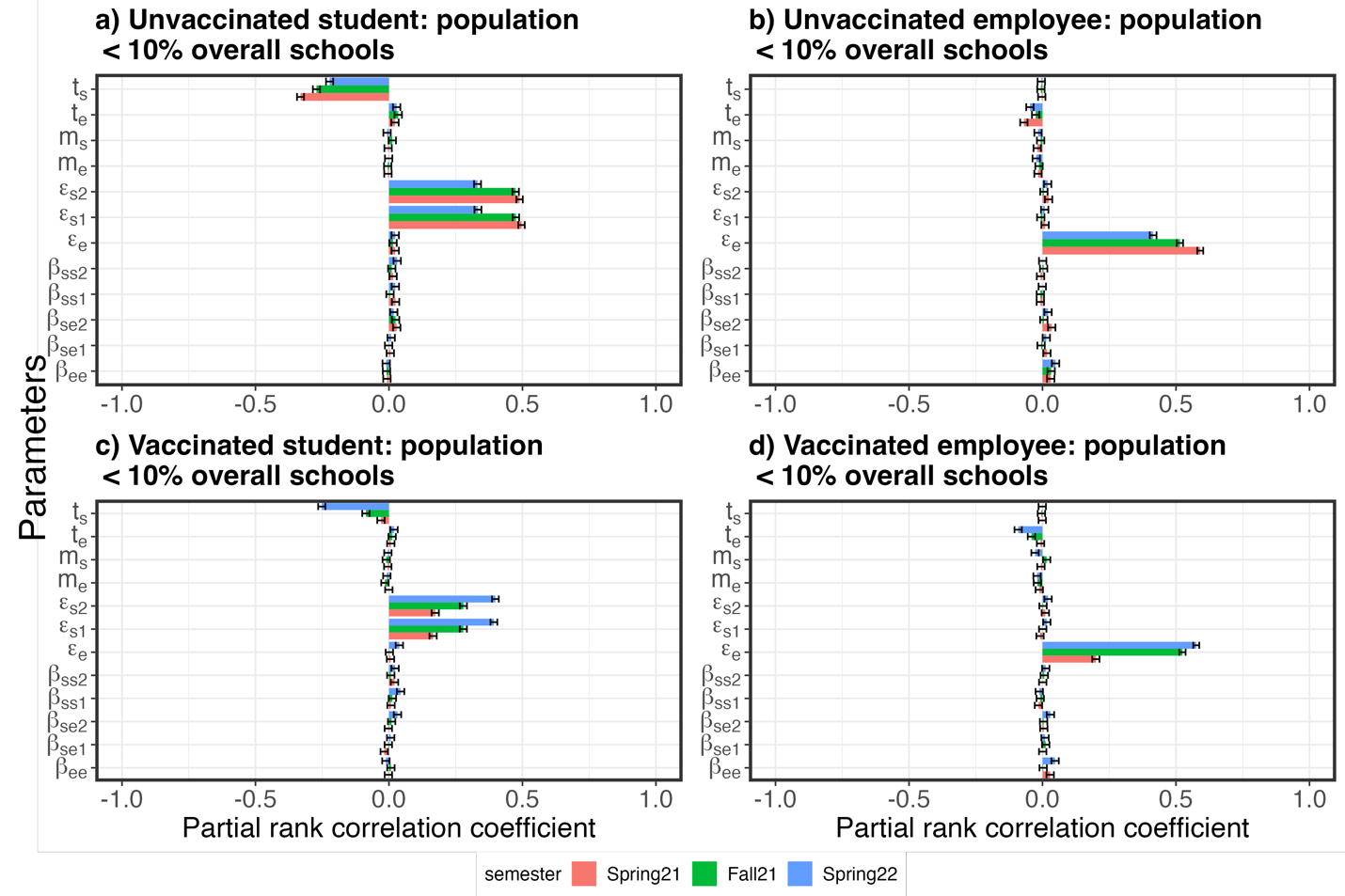


**Figure S6**, PRCCs of the model parameters on incidence risks when testing took place on each weekday per week in unvaccinated and vaccinated students (a and c) and employees (b and d) in school districts with population under 10% of overall school population during Spring 2021 (Pink), Fall 2021 (Green), and Spring 2022 (Blue). The bars are the medians, and the ticks are 95% confidence intervals.


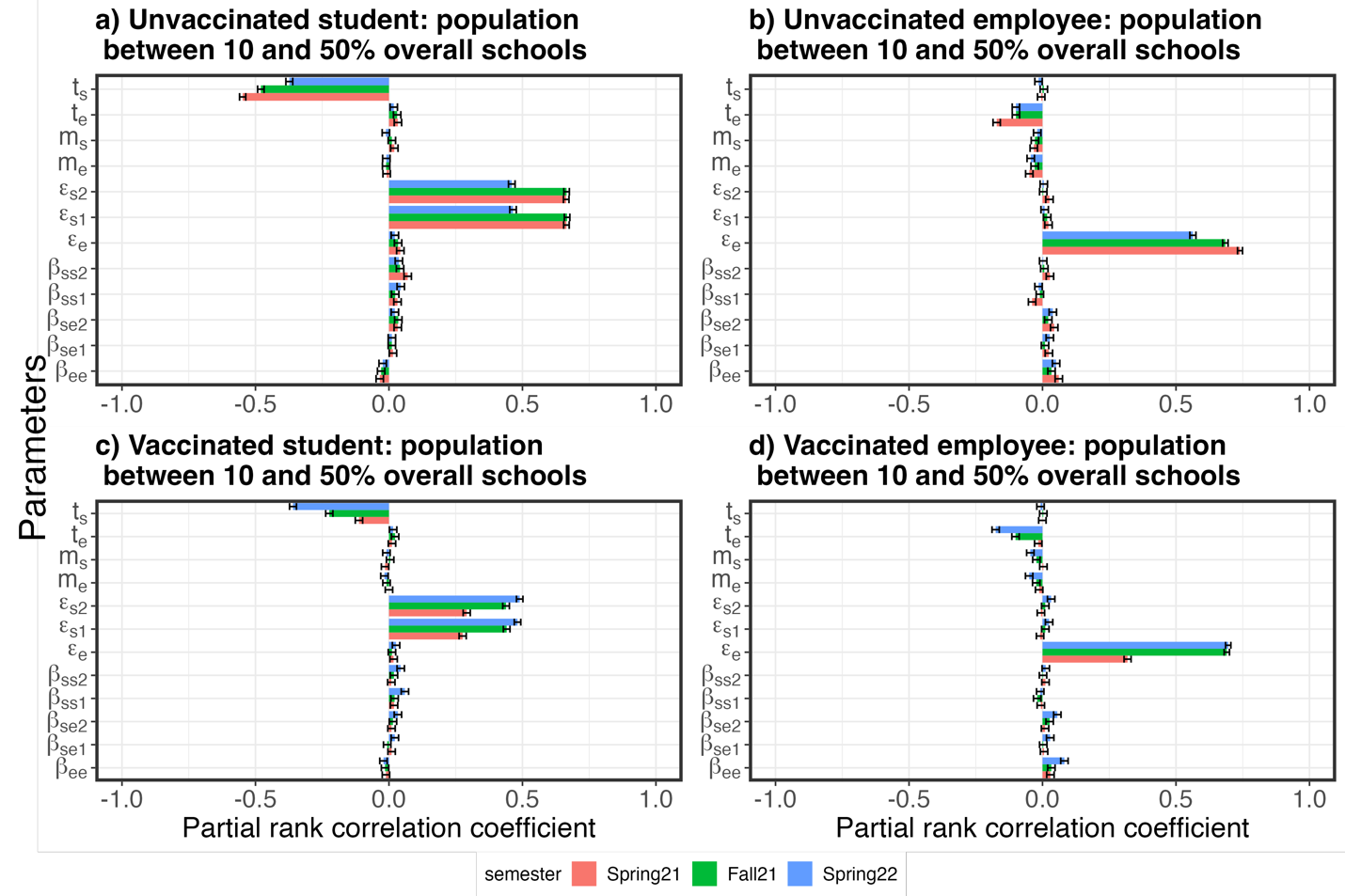


**Figure S7**, PRCCs of the model parameters on incidence risks when testing took place on each weekday per week in unvaccinated and vaccinated students (a and c) and employees (b and d) in school districts with population between 10 and 50% of overall school population during Spring 2021 (Pink), Fall 2021 (Green), and Spring 2022 (Blue). The bars are the medians, and the ticks are 95% confidence intervals.


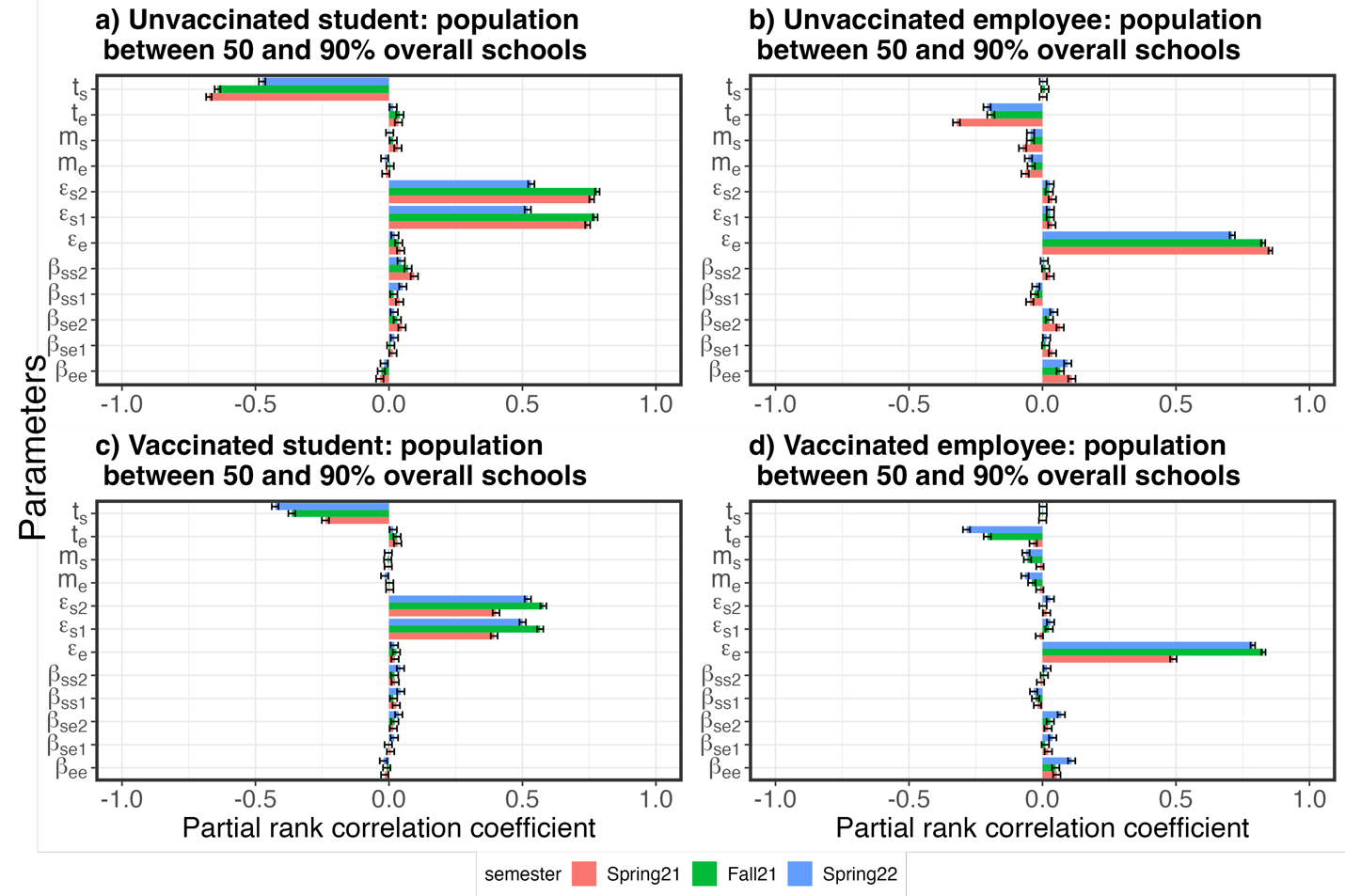


**Figure S8**, PRCCs of the model parameters on incidence risks when testing took place on each weekday per week in unvaccinated and vaccinated students (a and c) and employees (b and d) in school districts with population between 50 to 90% of overall school population during Spring 2021 (Pink), Fall 2021 (Green), and Spring 2022 (Blue). The bars are the medians, and the ticks are 95% confidence intervals.


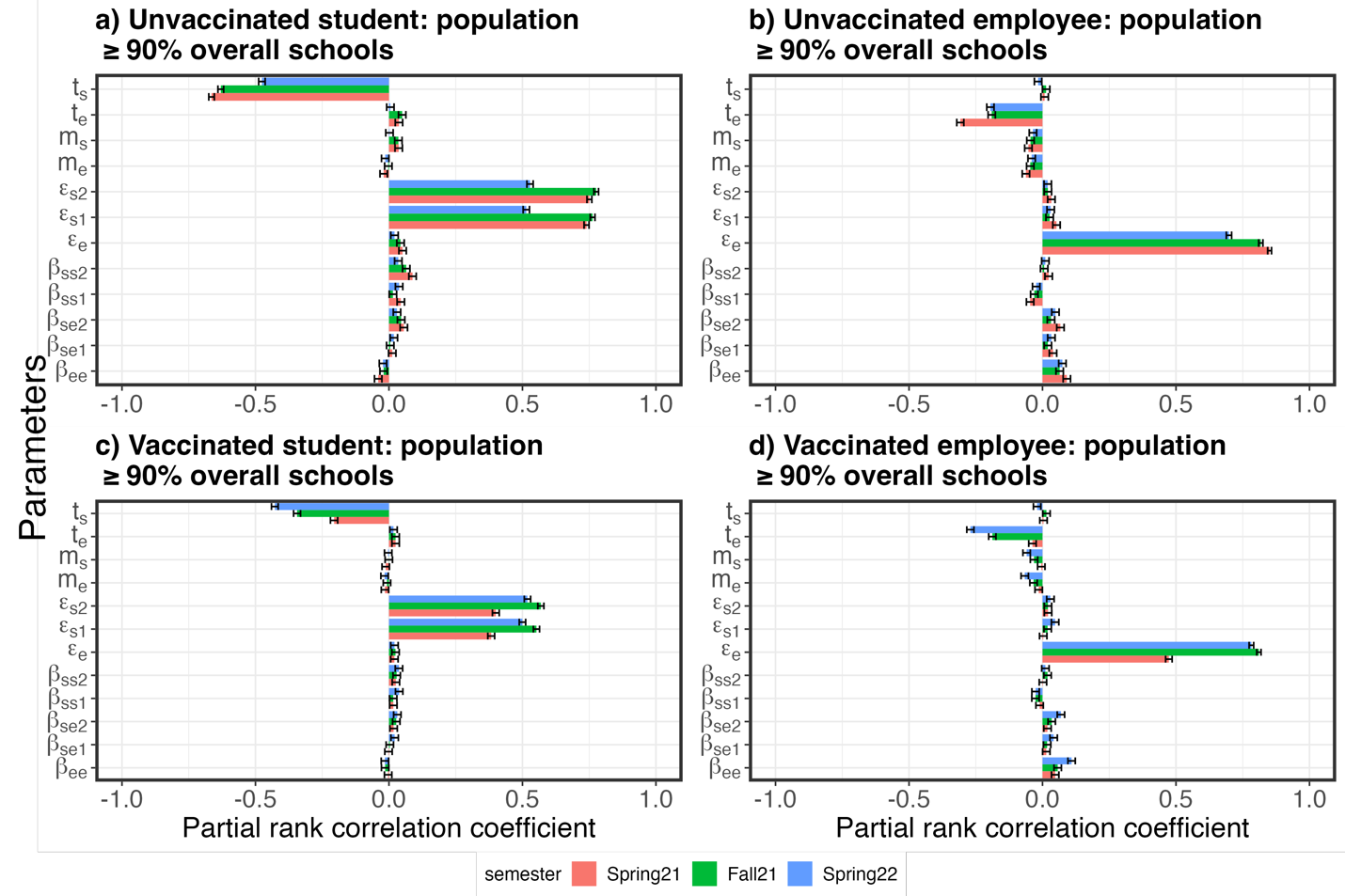


**Figure S9**, PRCCs of the model parameters on incidence risks when testing took place on each weekday per week in unvaccinated and vaccinated students (a and c) and employees (b and d) in school districts with population greater than 90% of overall school population during Spring 2021 (Pink), Fall 2021 (Green), and Spring 2022 (Blue). The bars are the medians, and the ticks are 95% confidence intervals.

**Table S1**, Corresponding PRCCs of model parameters from a global sensitivity analysis (**Figure 7**) on SARS-CoV-2 incidence risks in unvaccinated students (a and c) and employees (b and d) in the studied primary and middle school districts with populations below 10% (a and b) and between 50 and 90% (c and d) of the overall schools population.

| Model parameters | Parameter | PRCC estimation | PRCC 5% percentile | PRCC 95% percentile | Semester | Figure 7 |
| --- | --- | --- | --- | --- | --- | --- |
| Transmission among students in kindergarten to 5^th^ grade | $\beta_{ss1}$ | 0.03357007 | 0.01971732 | 0.04742283 | Fall21 | a |
| Transmission among students in 6^th^ to 8^th^ grade | $\beta_{ss2}$ | 0.01784628 | 0.00398792 | 0.03170464 |  |  |
| Transmission between employee and students in kindergarten to 5^th^ grade | $\beta_{se1}$ | -0.008037 | -0.0218971 | 0.00582314 |  |  |
| Transmission between employee and students in 6^th^ to 8^th^ grade | $\beta_{se2}$ | 0.00725857 | -0.0066016 | 0.02111877 |  |  |
| Transmission among employees | $\beta_{ee}$ | -0.0026527 | -0.0165132 | 0.01120782 |  |  |
| External transmission to students in kindergarten to 5^th^ grade | $\varepsilon_{s1}$ | 0.47205359 | 0.45983453 | 0.48427266 |  |  |
| External transmission to students in 6^th^ to 8^th^ grade | $\varepsilon_{s2}$ | 0.46656557 | 0.45430608 | 0.47882506 |  |  |
| External transmission to employee | $\varepsilon_{e}$ | 0.01482219 | 0.00096315 | 0.02868124 |  |  |
| Student mask adherence | $m_{s}$ | -0.0183549 | -0.0322131 | -0.0044966 |  |  |
| Employee mask adherence | $m_{e}$ | 0.00488885 | -0.0089716 | 0.01874925 |  |  |
| Student testing proportion | $T_{s}$ | -0.0861698 | -0.0999789 | -0.0723608 |  |  |
| Employee testing proportion | $T_{e}$ | 0.02059828 | 0.00674065 | 0.03445591 |  |  |
| Transmission among students in kindergarten to 5^th^ grade | $\beta_{ss1}$ | 0.054047 | 0.04020669 | 0.06788731 | Spring21 | a |
| Transmission among students in 6^th^ to 8^th^ grade | $\beta_{ss2}$ | 0.06165675 | 0.04782255 | 0.07549095 |  |  |
| Transmission between employee and students in kindergarten to 5^th^ grade | $\beta_{se1}$ | 0.00806086 | -0.0057993 | 0.02192098 |  |  |
| Transmission between employee and students in 6^th^ to 8^th^ grade | $\beta_{se2}$ | 0.01675841 | 0.00289979 | 0.03061704 |  |  |
| Transmission among employees | $\beta_{ee}$ | -0.0208514 | -0.034709 | -0.0069938 |  |  |
| External transmission to students in kindergarten to 5^th^ grade | $\varepsilon_{s1}$ | 0.47831944 | 0.46614728 | 0.49049159 |  |  |
| External transmission to students in 6^th^ to 8^th^ grade | $\varepsilon_{s2}$ | 0.47627297 | 0.4640854 | 0.48846053 |  |  |
| External transmission to employee | $\varepsilon_{e}$ | 0.04111732 | 0.02726847 | 0.05496617 |  |  |
| Student mask adherence | $m_{s}$ | -0.0189138 | -0.0327719 | -0.0050557 |  |  |
| Employee mask adherence | $m_{e}$ | -0.0015254 | -0.0153859 | 0.01233518 |  |  |
| Student testing proportion | $T_{s}$ | -0.11933 | -0.1330915 | -0.1055684 |  |  |
| Employee testing proportion | $T_{e}$ | 0.01557989 | 0.001721 | 0.02943878 |  |  |
| Transmission among students in kindergarten to 5^th^ grade | $\beta_{ss1}$ | 0.04755474 | 0.03370985 | 0.06139963 | Spring22 | a |
| Transmission among students in 6^th^ to 8^th^ grade | $\beta_{ss2}$ | 0.04194868 | 0.02810032 | 0.05579705 |  |  |
| Transmission between employee and students in kindergarten to 5^th^ grade | $\beta_{se1}$ | -0.000878 | -0.0147385 | 0.01298261 |  |  |
| Transmission between employee and students in 6^th^ to 8^th^ grade | $\beta_{se2}$ | 0.02543439 | 0.01157831 | 0.03929048 |  |  |
| Transmission among employees | $\beta_{ee}$ | -0.0097283 | -0.0235882 | 0.00413161 |  |  |
| External transmission to students in kindergarten to 5^th^ grade | $\varepsilon_{s1}$ | 0.32473952 | 0.31163014 | 0.3378489 |  |  |
| External transmission to students in 6^th^ to 8^th^ grade | $\varepsilon_{s2}$ | 0.32449641 | 0.31138588 | 0.33760694 |  |  |
| External transmission to employee | $\varepsilon_{e}$ | 0.02931945 | 0.01546484 | 0.04317407 |  |  |
| Student mask adherence | $m_{s}$ | -0.0221608 | -0.0360179 | -0.0083036 |  |  |
| Employee mask adherence | $m_{e}$ | -0.0145036 | -0.0283627 | -0.0006444 |  |  |
| Student testing proportion | $T_{s}$ | -0.0702058 | -0.0840321 | -0.0563794 |  |  |
| Employee testing proportion | $T_{e}$ | 0.01387778 | 1.85E-05 | 0.02773702 |  |  |
| Transmission among students in kindergarten to 5^th^ grade | $\beta_{ss1}$ | 0.00665977 | -0.0072005 | 0.02052003 | Fall21 | b |
| Transmission among students in 6^th^ to 8^th^ grade | $\beta_{ss2}$ | -0.0111608 | -0.0250205 | 0.00269894 |  |  |
| Transmission between employee and students in kindergarten to 5^th^ grade | $\beta_{se1}$ | 0.00255958 | -0.0113009 | 0.01642011 |  |  |
| Transmission between employee and students in 6^th^ to 8^th^ grade | $\beta_{se2}$ | 0.01390018 | 4.10E-05 | 0.02775941 |  |  |
| Transmission among employees | $\beta_{ee}$ | 0.02633982 | 0.01248406 | 0.04019558 |  |  |
| External transmission to students in kindergarten to 5^th^ grade | $\varepsilon_{s1}$ | 0.01814502 | 0.00428673 | 0.03200331 |  |  |
| External transmission to students in 6^th^ to 8^th^ grade | $\varepsilon_{s2}$ | 0.01052495 | -0.0033349 | 0.02438475 |  |  |
| External transmission to employee | $\varepsilon_{e}$ | 0.49673008 | 0.48470042 | 0.50875974 |  |  |
| Student mask adherence | $m_{s}$ | -0.0085542 | -0.0224142 | 0.00530591 |  |  |
| Employee mask adherence | $m_{e}$ | -0.0242675 | -0.038124 | -0.010411 |  |  |
| Student testing proportion | $T_{s}$ | 0.00085673 | -0.0130038 | 0.01471729 |  |  |
| Employee testing proportion | $T_{e}$ | -0.0104364 | -0.0242963 | 0.00342337 |  |  |
| Transmission among students in kindergarten to 5^th^ grade | $\beta_{ss1}$ | -0.0059976 | -0.0198579 | 0.00786274 | Spring21 | b |
| Transmission among students in 6^th^ to 8^th^ grade | $\beta_{ss2}$ | -5.11E-05 | -0.0139116 | 0.01380951 |  |  |
| Transmission between employee and students in kindergarten to 5^th^ grade | $\beta_{se1}$ | 0.01158111 | -0.0022785 | 0.02544075 |  |  |
| Transmission between employee and students in 6^th^ to 8^th^ grade | $\beta_{se2}$ | 0.04190757 | 0.02805918 | 0.05575596 |  |  |
| Transmission among employees | $\beta_{ee}$ | 0.02884735 | 0.01499255 | 0.04270215 |  |  |
| External transmission to students in kindergarten to 5^th^ grade | $\varepsilon_{s1}$ | 0.00805349 | -0.0058066 | 0.02191361 |  |  |
| External transmission to students in 6^th^ to 8^th^ grade | $\varepsilon_{s2}$ | 0.02650487 | 0.01264917 | 0.04036057 |  |  |
| External transmission to employee | $\varepsilon_{e}$ | 0.56545826 | 0.55402638 | 0.57689014 |  |  |
| Student mask adherence | $m_{s}$ | -0.0050925 | -0.0189529 | 0.00876793 |  |  |
| Employee mask adherence | $m_{e}$ | -0.0363789 | -0.0502303 | -0.0225275 |  |  |
| Student testing proportion | $T_{s}$ | -0.0015427 | -0.0154032 | 0.0123179 |  |  |
| Employee testing proportion | $T_{e}$ | -0.0159096 | -0.0297684 | -0.0020508 |  |  |
| Transmission among students in kindergarten to 5^th^ grade | $\beta_{ss1}$ | 0.01295135 | -0.0009081 | 0.02681076 | Spring22 | b |
| Transmission among students in 6^th^ to 8^th^ grade | $\beta_{ss2}$ | -0.0074056 | -0.0212658 | 0.00645458 |  |  |
| Transmission between employee and students in kindergarten to 5^th^ grade | $\beta_{se1}$ | 0.0148246 | 0.00096556 | 0.02868365 |  |  |
| Transmission between employee and students in 6^th^ to 8^th^ grade | $\beta_{se2}$ | 0.03188022 | 0.0180267 | 0.04573375 |  |  |
| Transmission among employees | $\beta_{ee}$ | 0.05819618 | 0.04435911 | 0.07203326 |  |  |
| External transmission to students in kindergarten to 5^th^ grade | $\varepsilon_{s1}$ | 0.01394574 | 8.65E-05 | 0.02780497 |  |  |
| External transmission to students in 6^th^ to 8^th^ grade | $\varepsilon_{s2}$ | 0.0102242 | -0.0036356 | 0.02408404 |  |  |
| External transmission to employee | $\varepsilon_{e}$ | 0.40704487 | 0.39438451 | 0.41970523 |  |  |
| Student mask adherence | $m_{s}$ | -0.0064804 | -0.0203407 | 0.00737989 |  |  |
| Employee mask adherence | $m_{e}$ | -0.0307231 | -0.0445772 | -0.0168691 |  |  |
| Student testing proportion | $T_{s}$ | 0.00018752 | -0.013673 | 0.01404809 |  |  |
| Employee testing proportion | $T_{e}$ | -0.0114939 | -0.0253536 | 0.0023657 |  |  |
| Transmission among students in kindergarten to 5^th^ grade | $\beta_{ss1}$ | 0.06822825 | 0.05439997 | 0.08205652 | Fall21 | c |
| Transmission among students in 6^th^ to 8^th^ grade | $\beta_{ss2}$ | 0.10527941 | 0.09149587 | 0.11906296 |  |  |
| Transmission between employee and students in kindergarten to 5^th^ grade | $\beta_{se1}$ | -0.0023917 | -0.0162522 | 0.01146887 |  |  |
| Transmission between employee and students in 6^th^ to 8^th^ grade | $\beta_{se2}$ | 0.02741314 | 0.01355778 | 0.0412685 |  |  |
| Transmission among employees | $\beta_{ee}$ | -0.0208796 | -0.0347371 | -0.007022 |  |  |
| External transmission to students in kindergarten to 5^th^ grade | $\varepsilon_{s1}$ | 0.76161948 | 0.75263749 | 0.77060146 |  |  |
| External transmission to students in 6^th^ to 8^th^ grade | $\varepsilon_{s2}$ | 0.76787874 | 0.75899973 | 0.77675776 |  |  |
| External transmission to employee | $\varepsilon_{e}$ | 0.04291234 | 0.02906453 | 0.05676014 |  |  |
| Student mask adherence | $m_{s}$ | -0.0276936 | -0.0415488 | -0.0138383 |  |  |
| Employee mask adherence | $m_{e}$ | 0.03393437 | 0.02008178 | 0.04778695 |  |  |
| Student testing proportion | $T_{s}$ | -0.2873754 | -0.3006513 | -0.2740995 |  |  |
| Employee testing proportion | $T_{e}$ | 0.05177483 | 0.03793285 | 0.06561681 |  |  |
| Transmission among students in kindergarten to 5^th^ grade | $\beta_{ss1}$ | 0.12323544 | 0.10948052 | 0.13699036 | Spring21 | c |
| Transmission among students in 6^th^ to 8^th^ grade | $\beta_{ss2}$ | 0.16940325 | 0.15574301 | 0.18306349 |  |  |
| Transmission between employee and students in kindergarten to 5^th^ grade | $\beta_{se1}$ | 0.00229149 | -0.011569 | 0.01615203 |  |  |
| Transmission between employee and students in 6^th^ to 8^th^ grade | $\beta_{se2}$ | 0.0293869 | 0.01553231 | 0.04324148 |  |  |
| Transmission among employees | $\beta_{ee}$ | -0.038126 | -0.0519765 | -0.0242755 |  |  |
| External transmission to students in kindergarten to 5^th^ grade | $\varepsilon_{s1}$ | 0.73022561 | 0.72075598 | 0.73969524 |  |  |
| External transmission to students in 6^th^ to 8^th^ grade | $\varepsilon_{s2}$ | 0.73916946 | 0.7298341 | 0.74850482 |  |  |
| External transmission to employee | $\varepsilon_{e}$ | 0.08664029 | 0.07283184 | 0.10044874 |  |  |
| Student mask adherence | $m_{s}$ | -0.0579761 | -0.0718134 | -0.0441389 |  |  |
| Employee mask adherence | $m_{e}$ | 0.02651247 | 0.01265677 | 0.04036817 |  |  |
| Student testing proportion | $T_{s}$ | -0.3369975 | -0.3500473 | -0.3239477 |  |  |
| Employee testing proportion | $T_{e}$ | 0.05961193 | 0.04577601 | 0.07344785 |  |  |
| Transmission among students in kindergarten to 5^th^ grade | $\beta_{ss1}$ | 0.09449963 | 0.08070108 | 0.10829817 | Spring22 | c |
| Transmission among students in 6^th^ to 8^th^ grade | $\beta_{ss2}$ | 0.07725945 | 0.06344031 | 0.09107859 |  |  |
| Transmission between employee and students in kindergarten to 5^th^ grade | $\beta_{se1}$ | 0.01461067 | 0.00075158 | 0.02846976 |  |  |
| Transmission between employee and students in 6^th^ to 8^th^ grade | $\beta_{se2}$ | 0.03973019 | 0.02588057 | 0.05357982 |  |  |
| Transmission among employees | $\beta_{ee}$ | -0.0081645 | -0.0220247 | 0.00569556 |  |  |
| External transmission to students in kindergarten to 5^th^ grade | $\varepsilon_{s1}$ | 0.50462056 | 0.49265416 | 0.51658696 |  |  |
| External transmission to students in 6^th^ to 8^th^ grade | $\varepsilon_{s2}$ | 0.50946834 | 0.49754146 | 0.52139521 |  |  |
| External transmission to employee | $\varepsilon_{e}$ | 0.03878745 | 0.02493731 | 0.05263759 |  |  |
| Student mask adherence | $m_{s}$ | -0.0306464 | -0.0445005 | -0.0167923 |  |  |
| Employee mask adherence | $m_{e}$ | -0.0048243 | -0.0186847 | 0.00903609 |  |  |
| Student testing proportion | $T_{s}$ | -0.2077793 | -0.2213373 | -0.1942212 |  |  |
| Employee testing proportion | $T_{e}$ | 0.01907041 | 0.00521236 | 0.03292846 |  |  |
| Transmission among students in kindergarten to 5^th^ grade | $\beta_{ss1}$ | -0.0026938 | -0.0165544 | 0.01116667 | Fall21 | d |
| Transmission among students in 6^th^ to 8^th^ grade | $\beta_{ss2}$ | 0.01156034 | -0.0022993 | 0.02541998 |  |  |
| Transmission between employee and students in kindergarten to 5^th^ grade | $\beta_{se1}$ | 0.0189946 | 0.00513653 | 0.03285267 |  |  |
| Transmission between employee and students in 6^th^ to 8^th^ grade | $\beta_{se2}$ | 0.05670709 | 0.04286883 | 0.07054536 |  |  |
| Transmission among employees | $\beta_{ee}$ | 0.07430972 | 0.06048747 | 0.08813197 |  |  |
| External transmission to students in kindergarten to 5^th^ grade | $\varepsilon_{s1}$ | 0.01464724 | 0.00078816 | 0.02850632 |  |  |
| External transmission to students in 6^th^ to 8^th^ grade | $\varepsilon_{s2}$ | 0.03872326 | 0.02487309 | 0.05257344 |  |  |
| External transmission to employee | $\varepsilon_{e}$ | 0.80766533 | 0.79949257 | 0.8158381 |  |  |
| Student mask adherence | $m_{s}$ | -0.0438657 | -0.0577129 | -0.0300185 |  |  |
| Employee mask adherence | $m_{e}$ | -0.047078 | -0.0609232 | -0.0332328 |  |  |
| Student testing proportion | $T_{s}$ | -0.0010736 | -0.0149342 | 0.01278696 |  |  |
| Employee testing proportion | $T_{e}$ | -0.0468765 | -0.0607218 | -0.0330311 |  |  |
| Transmission among students in kindergarten to 5^th^ grade | $\beta_{ss1}$ | -0.0254428 | -0.0392989 | -0.0115867 | Spring21 | d |
| Transmission among students in 6^th^ to 8^th^ grade | $\beta_{ss2}$ | 0.02797066 | 0.01411551 | 0.04182581 |  |  |
| Transmission between employee and students in kindergarten to 5^th^ grade | $\beta_{se1}$ | 0.03916015 | 0.02531021 | 0.05301009 |  |  |
| Transmission between employee and students in 6^th^ to 8^th^ grade | $\beta_{se2}$ | 0.10873346 | 0.09495507 | 0.12251185 |  |  |
| Transmission among employees | $\beta_{ee}$ | 0.11219557 | 0.09842252 | 0.12596863 |  |  |
| External transmission to students in kindergarten to 5^th^ grade | $\varepsilon_{s1}$ | 0.03174636 | 0.01789278 | 0.04559995 |  |  |
| External transmission to students in 6^th^ to 8^th^ grade | $\varepsilon_{s2}$ | 0.06026157 | 0.04642619 | 0.07409695 |  |  |
| External transmission to employee | $\varepsilon_{e}$ | 0.83646358 | 0.82886768 | 0.84405949 |  |  |
| Student mask adherence | $m_{s}$ | -0.0499074 | -0.0637507 | -0.0360641 |  |  |
| Employee mask adherence | $m_{e}$ | -0.0800624 | -0.0938784 | -0.0662463 |  |  |
| Student testing proportion | $T_{s}$ | 0.00737667 | -0.0064835 | 0.02123686 |  |  |
| Employee testing proportion | $T_{e}$ | -0.0967372 | -0.1105328 | -0.0829417 |  |  |
| Transmission among students in kindergarten to 5^th^ grade | $\beta_{ss1}$ | -0.0091967 | -0.0230567 | 0.00466325 | Spring22 | d |
| Transmission among students in 6^th^ to 8^th^ grade | $\beta_{ss2}$ | 0.01493345 | 0.00107443 | 0.02879248 |  |  |
| Transmission between employee and students in kindergarten to 5^th^ grade | $\beta_{se1}$ | 0.031199 | 0.01734518 | 0.04505283 |  |  |
| Transmission between employee and students in 6^th^ to 8^th^ grade | $\beta_{se2}$ | 0.08001417 | 0.06619804 | 0.09383029 |  |  |
| Transmission among employees | $\beta_{ee}$ | 0.1051147 | 0.09133091 | 0.11889848 |  |  |
| External transmission to students in kindergarten to 5^th^ grade | $\varepsilon_{s1}$ | 0.02039799 | 0.0065403 | 0.03425567 |  |  |
| External transmission to students in 6^th^ to 8^th^ grade | $\varepsilon_{s2}$ | 0.04450753 | 0.03066069 | 0.05835436 |  |  |
| External transmission to employee | $\varepsilon_{e}$ | 0.678724 | 0.66854489 | 0.68890311 |  |  |
| Student mask adherence | $m_{s}$ | -0.0439638 | -0.057811 | -0.0301167 |  |  |
| Employee mask adherence | $m_{e}$ | -0.0667945 | -0.0806241 | -0.0529649 |  |  |
| Student testing proportion | $T_{s}$ | -0.0100639 | -0.0239238 | 0.00379593 |  |  |
| Employee testing proportion | $T_{e}$ | -0.0754741 | -0.0892952 | -0.0616531 |  |  |

**Table S2**, Corresponding PRCCs of model parameters from a global sensitivity analysis (**Figure 9**) on SARS-CoV-2 incidence risks in unvaccinated students (a and c) and employees (b and d) in the studied primary and middle school districts with populations below 10% (a and b) and between 50 and 90% (c and d) of the overall schools population.

| Model parameters | Parameter | PRCC estimation | PRCC 5% percentile | PRCC 95% percentile | Academic semester | Figure 9 |
| --- | --- | --- | --- | --- | --- | --- |
| Transmission among students in kindergarten to 5^th^ grade | $\beta_{ss1}$ | 0.00386973 | -0.0099907 | 0.0177302 | Fall21 | a |
| Transmission among students in 6^th^ to 8^th^ grade | $\beta_{ss2}$ | 0.01053276 | -0.003327 | 0.02439256 |  |  |
| Transmission between employee and students in kindergarten to 5^th^ grade | $\beta_{se1}$ | -0.0013428 | -0.0152033 | 0.0125178 |  |  |
| Transmission between employee and students in 6^th^ to 8^th^ grade | $\beta_{se2}$ | 0.02483955 | 0.01098326 | 0.03869585 |  |  |
| Transmission among employees | $\beta_{ee}$ | -0.0091845 | -0.0230445 | 0.00467549 |  |  |
| External transmission to students in kindergarten to 5^th^ grade | $\varepsilon_{s1}$ | 0.47546298 | 0.46326934 | 0.48765662 |  |  |
| External transmission to students in 6^th^ to 8^th^ grade | $\varepsilon_{s2}$ | 0.47450478 | 0.46230397 | 0.48670559 |  |  |
| External transmission to employee | $\varepsilon_{e}$ | 0.01516499 | 0.00130601 | 0.02902397 |  |  |
| Student mask adherence | $m_{s}$ | 0.0123863 | -0.0014732 | 0.02624581 |  |  |
| Employee mask adherence | $m_{e}$ | -0.0047676 | -0.018628 | 0.00909281 |  |  |
| Student testing proportion | $T_{s}$ | -0.2716979 | -0.285037 | -0.2583587 |  |  |
| Employee testing proportion | $T_{e}$ | 0.03475427 | 0.02090207 | 0.04860646 |  |  |
| Transmission among students in kindergarten to 5^th^ grade | $\beta_{ss1}$ | 0.02474109 | 0.01088476 | 0.03859742 | Spring21 | a |
| Transmission among students in 6^th^ to 8^th^ grade | $\beta_{ss2}$ | 0.01535207 | 0.00149313 | 0.02921101 |  |  |
| Transmission between employee and students in kindergarten to 5^th^ grade | $\beta_{se1}$ | 0.00497463 | -0.0088858 | 0.01883503 |  |  |
| Transmission between employee and students in 6^th^ to 8^th^ grade | $\beta_{se2}$ | 0.02910497 | 0.01525027 | 0.04295967 |  |  |
| Transmission among employees | $\beta_{ee}$ | -0.0076046 | -0.0214648 | 0.00625555 |  |  |
| External transmission to students in kindergarten to 5^th^ grade | $\varepsilon_{s1}$ | 0.49655604 | 0.484525 | 0.50858708 |  |  |
| External transmission to students in 6^th^ to 8^th^ grade | $\varepsilon_{s2}$ | 0.48926659 | 0.47717831 | 0.50135487 |  |  |
| External transmission to employee | $\varepsilon_{e}$ | 0.02336814 | 0.00951135 | 0.03722492 |  |  |
| Student mask adherence | $m_{s}$ | -0.0033161 | -0.0171766 | 0.01054438 |  |  |
| Employee mask adherence | $m_{e}$ | -0.0038867 | -0.0177472 | 0.00997377 |  |  |
| Student testing proportion | $T_{s}$ | -0.3312602 | -0.3443382 | -0.3181822 |  |  |
| Employee testing proportion | $T_{e}$ | 0.0225542 | 0.00869715 | 0.03641124 |  |  |
| Transmission among students in kindergarten to 5^th^ grade | $\beta_{ss1}$ | 0.02382712 | 0.00997048 | 0.03768375 | Spring22 | a |
| Transmission among students in 6^th^ to 8^th^ grade | $\beta_{ss2}$ | 0.03084715 | 0.01699317 | 0.04470112 |  |  |
| Transmission between employee and students in kindergarten to 5^th^ grade | $\beta_{se1}$ | 0.00826985 | -0.0055902 | 0.02212995 |  |  |
| Transmission between employee and students in 6^th^ to 8^th^ grade | $\beta_{se2}$ | 0.01794033 | 0.00408199 | 0.03179867 |  |  |
| Transmission among employees | $\beta_{ee}$ | -0.0103223 | -0.0241821 | 0.00353755 |  |  |
| External transmission to students in kindergarten to 5^th^ grade | $\varepsilon_{s1}$ | 0.33330277 | 0.32023475 | 0.34637079 |  |  |
| External transmission to students in 6^th^ to 8^th^ grade | $\varepsilon_{s2}$ | 0.33174157 | 0.31866592 | 0.34481722 |  |  |
| External transmission to employee | $\varepsilon_{e}$ | 0.02346288 | 0.00960613 | 0.03731964 |  |  |
| Student mask adherence | $m_{s}$ | -0.0071462 | -0.0210064 | 0.00671406 |  |  |
| Employee mask adherence | $m_{e}$ | -0.0012463 | -0.0151069 | 0.01261424 |  |  |
| Student testing proportion | $T_{s}$ | -0.2223773 | -0.2358908 | -0.2088638 |  |  |
| Employee testing proportion | $T_{e}$ | 0.02879883 | 0.01494401 | 0.04265366 |  |  |
| Transmission among students in kindergarten to 5^th^ grade | $\beta_{ss1}$ | -0.0081541 | -0.0220142 | 0.00570599 | Fall21 | b |
| Transmission among students in 6^th^ to 8^th^ grade | $\beta_{ss2}$ | 0.00425785 | -0.0096026 | 0.01811829 |  |  |
| Transmission between employee and students in kindergarten to 5^th^ grade | $\beta_{se1}$ | -0.005403 | -0.0192634 | 0.00845734 |  |  |
| Transmission between employee and students in 6^th^ to 8^th^ grade | $\beta_{se2}$ | 0.00492639 | -0.008934 | 0.01878679 |  |  |
| Transmission among employees | $\beta_{ee}$ | 0.03231368 | 0.01846035 | 0.04616701 |  |  |
| External transmission to students in kindergarten to 5^th^ grade | $\varepsilon_{s1}$ | -0.0065402 | -0.0204005 | 0.0073201 |  |  |
| External transmission to students in 6^th^ to 8^th^ grade | $\varepsilon_{s2}$ | 0.00521829 | -0.0086421 | 0.01907867 |  |  |
| External transmission to employee | $\varepsilon_{e}$ | 0.51479934 | 0.50291653 | 0.52668216 |  |  |
| Student mask adherence | $m_{s}$ | -0.007075 | -0.0209352 | 0.00678523 |  |  |
| Employee mask adherence | $m_{e}$ | -0.0131317 | -0.0269911 | 0.00072765 |  |  |
| Student testing proportion | $T_{s}$ | -0.005946 | -0.0198063 | 0.00791431 |  |  |
| Employee testing proportion | $T_{e}$ | -0.0257872 | -0.0396431 | -0.0119312 |  |  |
| Transmission among students in kindergarten to 5^th^ grade | $\beta_{ss1}$ | -0.008172 | -0.0220321 | 0.00568808 | Spring21 | b |
| Transmission among students in 6^th^ to 8^th^ grade | $\beta_{ss2}$ | -0.0074635 | -0.0213237 | 0.00639671 |  |  |
| Transmission between employee and students in kindergarten to 5^th^ grade | $\beta_{se1}$ | 0.01654829 | 0.00268961 | 0.03040696 |  |  |
| Transmission between employee and students in 6^th^ to 8^th^ grade | $\beta_{se2}$ | 0.03451506 | 0.02066274 | 0.04836737 |  |  |
| Transmission among employees | $\beta_{ee}$ | 0.03074126 | 0.01688724 | 0.04459528 |  |  |
| External transmission to students in kindergarten to 5^th^ grade | $\varepsilon_{s1}$ | 0.00963451 | -0.0042254 | 0.02349444 |  |  |
| External transmission to students in 6^th^ to 8^th^ grade | $\varepsilon_{s2}$ | 0.02275521 | 0.00889823 | 0.03661219 |  |  |
| External transmission to employee | $\varepsilon_{e}$ | 0.59077525 | 0.57959204 | 0.60195846 |  |  |
| Student mask adherence | $m_{s}$ | -0.0197994 | -0.0336572 | -0.0059415 |  |  |
| Employee mask adherence | $m_{e}$ | -0.0169134 | -0.030772 | -0.0030548 |  |  |
| Student testing proportion | $T_{s}$ | -0.0028628 | -0.0167233 | 0.01099771 |  |  |
| Employee testing proportion | $T_{e}$ | -0.069784 | -0.0836108 | -0.0559573 |  |  |
| Transmission among students in kindergarten to 5^th^ grade | $\beta_{ss1}$ | -0.0008851 | -0.0147456 | 0.01297549 | Spring22 | b |
| Transmission among students in 6^th^ to 8^th^ grade | $\beta_{ss2}$ | 0.00062856 | -0.013232 | 0.01448912 |  |  |
| Transmission between employee and students in kindergarten to 5^th^ grade | $\beta_{se1}$ | 0.01412156 | 0.00026238 | 0.02798075 |  |  |
| Transmission between employee and students in 6^th^ to 8^th^ grade | $\beta_{se2}$ | 0.0206322 | 0.00677458 | 0.03448982 |  |  |
| Transmission among employees | $\beta_{ee}$ | 0.04892133 | 0.03507736 | 0.0627653 |  |  |
| External transmission to students in kindergarten to 5^th^ grade | $\varepsilon_{s1}$ | 0.00825721 | -0.0056029 | 0.02211731 |  |  |
| External transmission to students in 6^th^ to 8^th^ grade | $\varepsilon_{s2}$ | 0.01927724 | 0.00541924 | 0.03313523 |  |  |
| External transmission to employee | $\varepsilon_{e}$ | 0.4144904 | 0.40187654 | 0.42710426 |  |  |
| Student mask adherence | $m_{s}$ | -0.0168133 | -0.0306719 | -0.0029546 |  |  |
| Employee mask adherence | $m_{e}$ | -0.0234614 | -0.0373181 | -0.0096046 |  |  |
| Student testing proportion | $T_{s}$ | -0.0045561 | -0.0184165 | 0.00930437 |  |  |
| Employee testing proportion | $T_{e}$ | -0.047113 | -0.0609582 | -0.0332678 |  |  |
| Transmission among students in kindergarten to 5^th^ grade | $\beta_{ss1}$ | 0.01781445 | 0.00395608 | 0.03167282 | Fall21 | c |
| Transmission among students in 6^th^ to 8^th^ grade | $\beta_{ss2}$ | 0.07144734 | 0.05762219 | 0.08527248 |  |  |
| Transmission between employee and students in kindergarten to 5^th^ grade | $\beta_{se1}$ | 0.00680103 | -0.0070592 | 0.02066128 |  |  |
| Transmission between employee and students in 6^th^ to 8^th^ grade | $\beta_{se2}$ | 0.03123478 | 0.01738098 | 0.04508859 |  |  |
| Transmission among employees | $\beta_{ee}$ | -0.0286566 | -0.0425115 | -0.0148017 |  |  |
| External transmission to students in kindergarten to 5^th^ grade | $\varepsilon_{s1}$ | 0.77275658 | 0.76395924 | 0.78155392 |  |  |
| External transmission to students in 6^th^ to 8^th^ grade | $\varepsilon_{s2}$ | 0.78027646 | 0.77160757 | 0.78894534 |  |  |
| External transmission to employee | $\varepsilon_{e}$ | 0.03658064 | 0.02272935 | 0.05043194 |  |  |
| Student mask adherence | $m_{s}$ | 0.01583252 | 0.00197368 | 0.02969135 |  |  |
| Employee mask adherence | $m_{e}$ | 0.004306 | -0.0095544 | 0.01816644 |  |  |
| Student testing proportion | $T_{s}$ | -0.6421697 | -0.6527947 | -0.6315447 |  |  |
| Employee testing proportion | $T_{e}$ | 0.04093509 | 0.02708614 | 0.05478404 |  |  |
| Transmission among students in kindergarten to 5^th^ grade | $\beta_{ss1}$ | 0.03994073 | 0.02609122 | 0.05379024 | Spring21 | c |
| Transmission among students in 6^th^ to 8^th^ grade | $\beta_{ss2}$ | 0.09514556 | 0.08134787 | 0.10894325 |  |  |
| Transmission between employee and students in kindergarten to 5^th^ grade | $\beta_{se1}$ | 0.01485136 | 0.00099232 | 0.0287104 |  |  |
| Transmission between employee and students in 6^th^ to 8^th^ grade | $\beta_{se2}$ | 0.04884808 | 0.03500406 | 0.0626921 |  |  |
| Transmission among employees | $\beta_{ee}$ | -0.0342978 | -0.0481502 | -0.0204454 |  |  |
| External transmission to students in kindergarten to 5^th^ grade | $\varepsilon_{s1}$ | 0.74438802 | 0.73513266 | 0.75364338 |  |  |
| External transmission to students in 6^th^ to 8^th^ grade | $\varepsilon_{s2}$ | 0.75933044 | 0.75031129 | 0.76834958 |  |  |
| External transmission to employee | $\varepsilon_{e}$ | 0.04388701 | 0.0300398 | 0.05773423 |  |  |
| Student mask adherence | $m_{s}$ | 0.03365754 | 0.01980482 | 0.04751025 |  |  |
| Employee mask adherence | $m_{e}$ | -0.0117557 | -0.0256153 | 0.00210394 |  |  |
| Student testing proportion | $T_{s}$ | -0.6744703 | -0.6847036 | -0.6642371 |  |  |
| Employee testing proportion | $T_{e}$ | 0.03565916 | 0.0218074 | 0.04951091 |  |  |
| Transmission among students in kindergarten to 5^th^ grade | $\beta_{ss1}$ | 0.05204614 | 0.03820436 | 0.06588793 | Spring22 | c |
| Transmission among students in 6^th^ to 8^th^ grade | $\beta_{ss2}$ | 0.04574374 | 0.03189768 | 0.0595898 |  |  |
| Transmission between employee and students in kindergarten to 5^th^ grade | $\beta_{se1}$ | 0.0204207 | 0.00656302 | 0.03427838 |  |  |
| Transmission between employee and students in 6^th^ to 8^th^ grade | $\beta_{se2}$ | 0.01968384 | 0.00582596 | 0.03354173 |  |  |
| Transmission among employees | $\beta_{ee}$ | -0.0180798 | -0.0319382 | -0.0042215 |  |  |
| External transmission to students in kindergarten to 5^th^ grade | $\varepsilon_{s1}$ | 0.51990291 | 0.50806286 | 0.53174296 |  |  |
| External transmission to students in 6^th^ to 8^th^ grade | $\varepsilon_{s2}$ | 0.53311168 | 0.52138502 | 0.54483835 |  |  |
| External transmission to employee | $\varepsilon_{e}$ | 0.02231954 | 0.00846242 | 0.03617666 |  |  |
| Student mask adherence | $m_{s}$ | 0.0027386 | -0.0111219 | 0.01659912 |  |  |
| Employee mask adherence | $m_{e}$ | -0.0158365 | -0.0296954 | -0.0019777 |  |  |
| Student testing proportion | $T_{s}$ | -0.4750815 | -0.487278 | -0.462885 |  |  |
| Employee testing proportion | $T_{e}$ | 0.01545894 | 0.00160002 | 0.02931785 |  |  |
| Transmission among students in kindergarten to 5^th^ grade | $\beta_{ss1}$ | -0.0302348 | -0.0440891 | -0.0163806 | Fall21 | d |
| Transmission among students in 6^th^ to 8^th^ grade | $\beta_{ss2}$ | 0.0118818 | -0.0019778 | 0.02574139 |  |  |
| Transmission between employee and students in kindergarten to 5^th^ grade | $\beta_{se1}$ | 0.01163464 | -0.002225 | 0.02549427 |  |  |
| Transmission between employee and students in 6^th^ to 8^th^ grade | $\beta_{se2}$ | 0.02583805 | 0.01198211 | 0.03969399 |  |  |
| Transmission among employees | $\beta_{ee}$ | 0.06640317 | 0.05257319 | 0.08023315 |  |  |
| External transmission to students in kindergarten to 5^th^ grade | $\varepsilon_{s1}$ | 0.02889695 | 0.01504217 | 0.04275174 |  |  |
| External transmission to students in 6^th^ to 8^th^ grade | $\varepsilon_{s2}$ | 0.02522816 | 0.011372 | 0.03908432 |  |  |
| External transmission to employee | $\varepsilon_{e}$ | 0.82653571 | 0.81873381 | 0.83433761 |  |  |
| Student mask adherence | $m_{s}$ | -0.045417 | -0.0592633 | -0.0315707 |  |  |
| Employee mask adherence | $m_{e}$ | -0.0424709 | -0.056319 | -0.0286229 |  |  |
| Student testing proportion | $T_{s}$ | 0.00922858 | -0.0046314 | 0.02308856 |  |  |
| Employee testing proportion | $T_{e}$ | -0.1934394 | -0.2070382 | -0.1798407 |  |  |
| Transmission among students in kindergarten to 5^th^ grade | $\beta_{ss1}$ | -0.0477433 | -0.0615881 | -0.0338985 | Spring21 | d |
| Transmission among students in 6^th^ to 8^th^ grade | $\beta_{ss2}$ | 0.02862203 | 0.01476714 | 0.04247692 |  |  |
| Transmission between employee and students in kindergarten to 5^th^ grade | $\beta_{se1}$ | 0.037363 | 0.0235121 | 0.05121389 |  |  |
| Transmission between employee and students in 6^th^ to 8^th^ grade | $\beta_{se2}$ | 0.06598992 | 0.05215956 | 0.07982028 |  |  |
| Transmission among employees | $\beta_{ee}$ | 0.10993543 | 0.09615887 | 0.12371199 |  |  |
| External transmission to students in kindergarten to 5^th^ grade | $\varepsilon_{s1}$ | 0.03500202 | 0.02114994 | 0.04885409 |  |  |
| External transmission to students in 6^th^ to 8^th^ grade | $\varepsilon_{s2}$ | 0.03686812 | 0.02301698 | 0.05071927 |  |  |
| External transmission to employee | $\varepsilon_{e}$ | 0.85351581 | 0.84629353 | 0.8607381 |  |  |
| Student mask adherence | $m_{s}$ | -0.0747146 | -0.0885364 | -0.0608927 |  |  |
| Employee mask adherence | $m_{e}$ | -0.065307 | -0.079138 | -0.051476 |  |  |
| Student testing proportion | $T_{s}$ | 0.00244159 | -0.0114189 | 0.01630212 |  |  |
| Employee testing proportion | $T_{e}$ | -0.3223147 | -0.3354356 | -0.3091938 |  |  |
| Transmission among students in kindergarten to 5^th^ grade | $\beta_{ss1}$ | -0.0249897 | -0.0388459 | -0.0111334 | Spring22 | d |
| Transmission among students in 6^th^ to 8^th^ grade | $\beta_{ss2}$ | 0.00677821 | -0.007082 | 0.02063846 |  |  |
| Transmission between employee and students in kindergarten to 5^th^ grade | $\beta_{se1}$ | 0.01527028 | 0.00141133 | 0.02912924 |  |  |
| Transmission between employee and students in 6^th^ to 8^th^ grade | $\beta_{se2}$ | 0.04246453 | 0.02861646 | 0.0563126 |  |  |
| Transmission among employees | $\beta_{ee}$ | 0.09449795 | 0.08069941 | 0.1082965 |  |  |
| External transmission to students in kindergarten to 5^th^ grade | $\varepsilon_{s1}$ | 0.02940873 | 0.01555415 | 0.0432633 |  |  |
| External transmission to students in 6^th^ to 8^th^ grade | $\varepsilon_{s2}$ | 0.02845207 | 0.01459711 | 0.04230703 |  |  |
| External transmission to employee | $\varepsilon_{e}$ | 0.71080568 | 0.70105632 | 0.72055505 |  |  |
| Student mask adherence | $m_{s}$ | -0.0447783 | -0.0586249 | -0.0309316 |  |  |
| Employee mask adherence | $m_{e}$ | -0.0527497 | -0.066591 | -0.0389085 |  |  |
| Student testing proportion | $T_{s}$ | 0.00321451 | -0.010646 | 0.01707501 |  |  |
| Employee testing proportion | $T_{e}$ | -0.2077164 | -0.2212747 | -0.1941582 |  |  |
